# Clinical expression and antigenic profiles of a *Plasmodium vivax* vaccine candidate: merozoite surface protein 7 (PvMSP-7)

**DOI:** 10.1186/s12936-019-2826-7

**Published:** 2019-06-13

**Authors:** Chew Weng Cheng, Somchai Jongwutiwes, Chaturong Putaporntip, Andrew P. Jackson

**Affiliations:** 1 Department of Infection Biology, Institute of Infection and Global Health, University of Liverpool, 146 Brownlow Hill, Liverpool, L3 5RF UK; 2 Molecular Biology of Malaria and Opportunistic Parasites Research Unit, Department of Parasitology, Faculty of Medicine, Chulalongkorn University, Bangkok, Thailand

**Keywords:** Malaria, *Plasmodium vivax*, PvMSP-7, Transcriptomics, Antigen, Epitope, Clinical isolates, Vaccine

## Abstract

**Background:**

Vivax malaria is the predominant form of malaria outside Africa, affecting about 14 million people worldwide, with about 2.5 billion people exposed. Development of a *Plasmodium vivax* vaccine is a priority, and merozoite surface protein 7 (MSP-7) has been proposed as a plausible candidate. The *P. vivax* genome contains 12 MSP-7 genes, which contribute to erythrocyte invasion during blood-stage infection. Previous analysis of MSP-7 sequence diversity suggested that not all paralogs are functionally equivalent. To explore MSP-7 functional diversity, and to identify the best vaccine candidate within the family, MSP-7 expression and antigenicity during bloodstream infections were examined directly from clinical isolates.

**Methods:**

Merozoite surface protein 7 gene expression was profiled using RNA-seq data from blood samples isolated from ten human patients with vivax malaria. Differential expression analysis and co-expression cluster analysis were used to relate PvMSP-7 expression to genetic markers of life cycle stage. Plasma from vivax malaria patients was also assayed using a custom peptide microarray to measure antibody responses against the coding regions of 12 MSP-7 paralogs.

**Results:**

Ten patients presented diverse transcriptional profiles that comprised four patient groups. Two MSP-7 paralogs, 7A and 7F, were expressed abundantly in all patients, while other MSP-7 genes were uniformly rare (e.g. 7J). MSP-7H and 7I were significantly more abundant in patient group 4 only, (two patients having experienced longer patency), and were co-expressed with a schizont-stage marker, while negatively associated with liver-stage and gametocyte-stage markers. Screening infections with a PvMSP-7 peptide array identified 13 linear B-cell epitopes in five MSP-7 paralogs that were recognized by plasma from all patients.

**Conclusions:**

These results show that MSP-7 family members vary in expression profile during blood infections; MSP-7A and 7F are expressed throughout the intraerythrocytic development cycle, while expression of other paralogs is focused on the schizont. This may reflect developmental regulation, and potentially functional differentiation, within the gene family. The frequency of B-cell epitopes among paralogs also varies, with MSP-7A and 7L consistently the most immunogenic. Thus, MSP-7 paralogs cannot be assumed to have equal potential as vaccines. This analysis of clinical infections indicates that the most abundant and immunogenic paralog is MSP-7A.

**Electronic supplementary material:**

The online version of this article (10.1186/s12936-019-2826-7) contains supplementary material, which is available to authorized users.

## Background

*Plasmodium vivax* is a human malaria parasite of global importance, predominantly confined to populations outside sub-Saharan Africa [1]. The biology of *P. vivax* differs to that of *Plasmodium falciparum*; although *P. vivax* can cause severe and fatal disease, most infections are mild or asymptomatic. Unlike *P. falciparum, P. vivax* can form a dormant liver stage, the hypnozoite, and relapse due to parasite re-emergence is thought to have a major role in the epidemiology of vivax malaria [2]. In Asia, almost half of malaria infections are caused by *P. vivax* [3] and its importance continues to increase with the emergence of chloroquine and sulfadoxine–pyrimethamine resistant strains [4]. Therefore, the burden of vivax malaria outside Africa must not be under-appreciated and new control strategies, including a vaccine, are needed to prevent disease. While many vaccine candidates have been identified, few have progressed beyond pre-clinical evaluation. Circumsporozoite protein (CSP), ookinete surface protein Pvs25 and Duffy binding protein (PvDBP_RII) each elicit strong antibody responses that inhibit infection but have not yet produced sterile protection [5–9]. Detailed studies are needed of these and other antigens expressed in the bloodstream, which could provide vaccines that block transmission, either to the human host or the vector [10].

Merozoite surface protein 7 (MSP-7) is a multigene family conserved across *Plasmodium* but varying in copy number among species. In *P. vivax*, MSP-7 consists of 12 paralogs (PvMSP7-A to -L), arranged in tandem at a single position on chromosome 12 [11–13]. All MSP-7 proteins are developmentally regulated and expressed on the merozoite surface during asexual blood stages [14]. MSP-7 forms a multi-protein complex with merozoite surface protein 1 (MSP-1) on red blood cell band 3 that facilitates initial invasion [15–17]. Knock-out of *Plasmodium berghei* MSP-7 in mouse reduces parasite propagation [18], while disruption of *P. falciparum* MSP-7 inhibits merozoite invasion [19]. Thus, MSP-7 collectively is thought to have a key role in host cell invasion, but it is unclear if all paralogs have equivalent roles in this general phenotype. Recent population genetic analysis has revealed consistent heterogeneity in allelic diversity among the 12 genes, with PvMSP-7B, C, E, G, H, and I displaying much higher genetic diversity compared to A and F [20–22], which suggests that the gene paralogs have evolved under different functional constraints.

Merozoite surface protein 7 is a plausible vaccine target due to its putative role in erythrocyte invasion [23, 24]. Given that the scale of functional differentiation among paralogs is unknown, it is important to consider variation in expression profile, molecular interactions, and immunogenicity within the gene family in order to make informed decisions about choice of vaccine candidates. There is some understanding of MSP-7 expression profiles from global transcriptomes of synchronized *P. vivax* batch cell cultures measured using microarray [25] and RNA sequencing [26]. These suggested that PvMSP-7 paralogs may display substantial variation in expression through the 48-h intraerythrocytic developmental cycle (IDC). In this study, variation in expression profiles among PvMSP-7 paralogs is examined using RNA-seq applied to asynchronous parasite populations isolated from ten clinical infections. Clustering analysis is used to associate PvMSP-7 paralogs with other developmentally-regulated genes to better understand functional differentiation within the family. Clustering genes based on their transcriptional profiles can provide functional information for uncharacterized genes through their associations [27]. This approach revealed a novel function in osmotic protection during merozoite and ring stage for Cytochrome *C* heme-lyase in *P. falciparum* [28], and also determined that a conserved *Plasmodium* protein, PF3D7_0418300, clustered robustly with MSP-9 and two AP2 domain transcription factors (SPE2-interacting protein and APIAP2), indicating that it has a role in transcriptional regulation [29].

The antibody responses of clinical isolates to a custom peptide microarray of all PvMSP-7 sequences are also examined to identify variation in immunogenicity among paralogs. Peptide microarrays have been used to characterize the immunogenicity of other malaria vaccine candidates. Nixon et al. [30] identified five immunoreactive epitopes in *P. falciparum* schizont egress antigen 1 (PfSEA-1A) using a 15-mer peptide microarray exposed to plasma from malaria patients in Kenya and showed these that antibody responses against these epitopes could produce a significant reduction of parasitaemia in vaccinated animals. Similarly, Quintana et al. [31] screened serum from infected children with a peptide microarray containing three *P. falciparum* gene families (PfEMP1, RIFIN, and SURFIN) implicated in severe malaria, and identified multiple reactive peptides. In *P. vivax*, Chootong et al. [32] used a microarray containing 178 peptides of the entire Duffy binding protein (PvDBP) to identify ten linear B-cell epitopes; these were located mostly in the central protein domain, consistent with previous observations that this is essential for receptor recognition [33]. Thus, mapping epitopes is a key stage in identifying vaccine candidates, and, by extension, where multi-copy gene families are concerned, evaluating epitopic variation among paralogs is also important.

This study advances the understanding of functional variation within the MSP-7 gene family, a vital component of the parasite cell surface during the asexual blood stages of *P. vivax* and other species. It shows that paralogs vary substantially in their abundance during the IDC and in their immunogenicity during clinical infections, and, consequently, it cannot be assumed that all MSP-7 proteins will make equally suitable antigens for experimental vaccines.

## Methods

### Human ethics statement

This work was approved by the Institutional Review Board for Human Research of the Faculty of Medicine, Chulalongkorn University, Thailand (IRB No. 104/59). Written consent was obtained from all participants or from their parents or guardians prior to admission into the study.

### Sample collection and processing

For transcriptomic analysis, five human patients were recruited from each of two malaria endemic areas of Thailand: (i) Ubon Ratchathani, located along the North-eastern border between Thailand and Cambodia, and (ii) Yala, located along the Southern border between Thailand and Malaysia. Patients of any age who attended malaria clinic with fever but did not show severe malaria symptoms were recruited to the study. Hence, all patients were symptomatic and were asked to provide information on the days of fever they had experienced (Table 1). Patients were screened with microscopic examination by an experienced laboratory technician and ~ 600 μL of venous blood were collected from *P. vivax*-infected patients. Blood samples were preserved in RNAlater^®^ solution (Ambion, Grand Island, NY, USA) with a 1:1 ratio and stored at − 20 °C until processing. Whole RNA was extracted from 500 μL of each blood sample using a QIAamp RNA blood mini kit (Qiagen, Hilden, Germany), following the manufacturer’s recommendations and stored at − 80 °C until used. A nested rRNA polymerase chain reaction (PCR) capable of detecting all five human malaria species [34] was used to confirm the presence of *P. vivax* and the absence of other species.

**Table 1:** Ten patients diagnosed with *P. vivax* infection were recruited in the study from two malaria clinics in Thailand: Ubon Ratchathani and Yala

Sample	Age (years)	Gender	Province	Days of patency	Parasitaemia (parasites/200 leukocytes)	Patient group
UBT3086	50	Male	Ubon Ratchathani	2	220	1
UBT3087	NR	NR	Ubon Ratchathani	3	152	2
UBT3089	41	Male	Ubon Ratchathani	2	60	2
UBT3090	14	Male	Ubon Ratchathani	2	226	2
UBT3091	37	Male	Ubon Ratchathani	3	383	1
YL3111	26	Female	Yala	2	360	3
YL3112	45	Male	Yala	2	20	3
YL3113	15	Male	Yala	7	160	4
YL3114	30	Female	Yala	2	40	3
YL3115	46	Female	Yala	7	360	4

Patients ranged between 14 and 50 years of age. Information regarding days of patency was provided by patients to medical officers during their visit

*NR* not recorded

For screening of the MSP-7 peptide microarray, plasma was collected from 64 vivax-malaria patients from Ubon Ratchathani province (North-eastern Thailand) between 2013 and 2016. Negative controls, i.e. malaria naïve donors (n = 20), were recruited from non-malaria endemic areas at Chulalongkorn hospital, Bangkok. These patients had not lived/spent time in malaria-endemic regions of Thailand, as far as was known. Approximately 2 mL of venous blood was collected into EDTA anticoagulant from patients with single-strain *P. vivax* infection, confirmed by nested rRNA PCR [34], and plasma was collected by centrifugation. These plasma were sorted into five experimental age cohorts: 0–14 years (n = 11), 15–29 years (n = 22), 30–44 (n = 19), 45–59 (n = 10) and 60–74 (n = 2). A master plasma sample was prepared for each age group by pooling 5 µL of plasma from each vivax-infected patient. All samples were preserved at − 80 °C until use.

### RNA sequencing

Ten RNA samples were treated with DNase, followed by Epicentre Globin-Zero Gold kit to deplete rRNA and globin transcripts. RNA-seq libraries were prepared at the Centre for Genomic Research, University of Liverpool using the NEBNext Ultra Directional protocol. The RNA-seq libraries were sequenced on the Illumina HiSeq4000 platform in a single lane, generating ~ 30 million paired-end 150 bp reads for each sample. The quality of the libraries was affirmed by Agilent 2100 Bioanalyser. RNA-seq data have been deposited in the EMBL-EBI ArrayExpress database (http://www.ebi.ac.uk/arrayexpress/experiments) under Accession Number E-MTAB-6753.

### Estimation of transcript abundance

The initial processing and quality assessment of the raw reads began with base calling and de-multiplexing of indexed reads using *CASAVA* version 1.8.2 (Illumina) to produce ten samples from one lane of sequence data in fastq format. The Illumina adapter sequences were trimmed from the raw fastq files by using *Cutadapt* version 1.2.1 [35]. The option “-O 3” was specified to trim off the 3′ end of any reads that matched to the adapter sequence over at least 3 bp. The reads were further trimmed to remove low quality bases, using *Sickle* version 1.2 with a minimum window quality score of 20. Before mapping the filtered reads to the reference genome, reads shorter than 10 bp were removed. Subsequently, all the filtered reads were mapped to the human genome (GRCH37) using *TopHat2* [36]. The unmapped reads were then aligned to the *PvP01* reference genome [37] using *TopHat2*. Following the alignment of reads to the *P. vivax* genome, the read counts of each gene were quantified with *featureCounts* [38]. Read counts were estimated by dividing the total number of mapped reads by the length of the gene, and then scaling the estimates to one million. The genome-wide coverage of each sample was determined using *Qualimap 2* [39]. Transcript abundance, expressed as Read Count per Million bases (CPM) was estimated by *DESeq2* [40]; this program normalizes data for both library size and compositional differences (e.g. the presence and absence of large abundance transcripts) using median ratios of log_2_-transformed raw counts. To establish reproducibility among the transcriptomes and to identify possible low-quality outliers, Pearson correlations of transcript abundances (N = 5838) were estimated for each pair of samples in *R* version 3.4.3 and *corrplot* [41].

### Differential gene expression analysis

Principal component analysis (PCA) was performed on the log-transformed, normalized CPM values for 5838 genes estimated by *DESeq2* for ten patients. The PCA consistently placed the ten samples into four groups, which were adopted subsequently to identify differentially expressed genes (DEGs) in a pairwise manner, using *DESeq2* [40]. Genes with a false discovery rate (FDR) threshold below 0.05 after correcting for multiple tests were deemed significantly differentially expressed. Additionally, differential expression analysis was also performed analysis with *EdgeR* [42], but the DEGs and PCA plot did not deviate substantially from the *DESeq2* results. Thus, the latter was used in downstream analyses.

### Co-expression analysis

The DEGs identified for each patient group were imported into *coseq*, an R package to identify clusters of co-expressed genes [43]. The purpose was to associate differentially-expressed PvMSP-7 genes with other genes that are known to be developmentally regulated. A robust association with a known life-stage marker would then allow developmental regulation within the PvMSP-7 family to be inferred. *Coseq* uses Gaussian mixture models to cluster all the genes based on the proportion of normalized counts in their expression profiles. The *DESeq2* data were fitted to a Gaussian mixture model on either arcsine- or logit-transformed normalized profiles. One hundred clusters were tested in each transformation with the following commands; arcsin_transformed < − coseq(counts, K = 2:100, model = “Normal”, transformation = “arcsin”) and logit_transformed < − coseq(counts, K = 2:100, model = “Normal”, transformation = “logit”). To choose accurately between two transformation models, *coseq* calculated the corrected integrated completed likelihood (ICL) values from these two models and selected the number of clusters and preferred model-transformation based on the highest corrected ICL value.

### Enrichment analysis and pathway identification

To infer the functional consequences of differential gene expression between patient groups, enrichment analysis was carried out on functional terms associated with the DEGs, using *PlasmoDB* release 35 [44]. First, heat-maps were plotted for the DEGs from each patient group comparison, relating Euclidian distance-based dendrograms of all DEG expression profiles and each patient group. These heat-maps neatly divided DEGs into clades based on their expression profiles and these were then subjected to enrichment analysis for both Gene Ontology (GO) terms [45] and Kyoto Encyclopaedia of Genes and Genomes (KEGG) pathways [46]. Functional terms and pathways were deemed significant where the adjusted *p*-value was < 0.05. Second, the genes belonging to clusters returned by *Coseq* were also subjected to the same enrichment analyses.

### Microarray screening

A custom peptide microarray was designed that included the entire, predicted amino acid sequences of all 13 PvMSP-7 paralogs. The microarray contained 15-mer peptides of each PvMSP-7 gene with an overlap of 11 amino acids, printed in duplicate: a total of 2346 peptides. The slides were manufactured by PepperPrint (Heidelberg, Germany) and coated with a poly(ethylene glycol)-based graft copolymer with a thickness of 13.5 nm and an additional three amino acid linker (β-alanine, aspartic acid, and β-alanine), which ensure optimal epitope orthogonal attachment and presentation. Each microarray slide consisted of three identical array copies, each framed by flag anti-HA (YPYCVPDYAG, 52 spots) as a quality control measurement. The signal intensities of these control peptides indicate the spot uniformity and binding specificities.

Each microarray was treated with 1% bovine serum albumin (BSA) blocking buffer for 30 min at room temperature. To determine the background intensity, one microarray was then incubated with goat anti-human IgG (H + L) DyLight680 antibody (Rockland Immunochemical Inc., USA) that was diluted 1:2500 in staining buffer (phosphate-buffered saline (PBS) with 0.05% Tween20 and 10% blocking buffer). Clean microarrays were then incubated overnight at 4 °C with 10 µL pooled plasma from one of five patient age cohorts (see above), diluted 1:50 with staining buffer. The peptide microarray was washed three times with standard buffer (PBS with 0.05% Tween20, pH 7.4) using an orbital shaker at 140 rpm. The microarrays were washed in 1 mM Tris dipping buffer (pH 7.4) to remove all contaminants from the array surface and dried by tapping the edge of the slide on tissue paper. To ensure that all microarrays were responding correctly, all steps were repeated with the Cy3-conjugated anti-HA control antibody supplied by PepperPrint (Heidelberg, Germany). A sixth microarray was assayed to provide a negative control consisting of naïve human plasma taken from individuals in malaria-free areas.

Microarrays were illuminated on an Agilent G2565CA microarray scanner, with the AgilentHD red colour single channel at 10 µm resolution and output to a 16-bit greyscale tiff files. The mean and median of local background and foreground fluorescence intensity values for all peptide spots were quantified using PepSlide analyser (SICASYS Software, Heidelberg, Germany). Correction for background intensity and normalization for variation between and within arrays were performed using the LIMMA package [47] implemented in R version 3.4.3. Background correction was performed on each array by subtraction of local background estimates from each foreground intensity value [48]. This step was performed to eliminate the effects of non-specific binding across the arrays. Spot intensities were also normalization for between-array variation using the quantile principle in LIMMA [49]. The quantile function compensates for systematic measurement errors between-arrays, producing a more uniform intensity distribution and leaving only the true biological differences in the datasets.

### Microarray statistical analysis

Statistical analysis was performed using the LIMMA package [47]. Normalized data were fitted to a linear model and peptides with significant responses to antibody, i.e. normalized fluorescent intensity significantly greater than background levels, were determined in all experimental groups by comparing the log transformation of expression intensity. A simple Bayesian model was used to estimate significant peptides between the experimental group and negative control [47, 49]. The summary statistics were computed by the eBayes() function, returning a list of significantly expressed peptides were displayed. Benjamini and Hochberg’s method or false discovery rate (FDR) was used to correct the p-value for multiple tests. FDR < 0.05 were considered statistically significant.

## Results

### Data summary

RNA sequencing generated ~ 30 million 150 bp paired-end reads for each of ten clinical isolates (Table 2). The samples were collected from field hospitals in remote areas and suffered a variable degree of degradation prior to sequencing; RIN values were consistent but low (between 2 and 2.8); moreover, sequence coverage was variable and relatively low for some isolates. For each isolate, all reads were aligned to the human genome (GRCh37) and then the unmapped reads were aligned to the *P. vivax* reference genome (PvP01; [37]). Typically, more than 70% of reads were derived from human, while reads mapping to *P. vivax* ranged between 0.78 and 22.38%. Mean genome coverage varied widely among the ten isolates, between 1.07× and 78.52× (mean = 18.8×). Isolate UBT3089 has the highest mean coverage of 78.52×, while five samples have a value < 10×. Global transcript abundance, expressed in Read Count per Million bases (CPM) and normalized for gene length and read number, was calculated for all genes using *DESeq2* after mapping the data to the reference genome with *TopHat2* (Additional file 1). To evaluate the reproducibility of the results in the light of this variation in coverage, Pearson correlation coefficients were calculated between each sample for gene-level CPM estimates; such coefficients ranged from 0.59 to 0.91 (mean = 0.72).

**Table 2:** Summary statistics of RNA preparation, RNA sequencing, read mapping to the human GRCh37 and *P. vivax* P01 genomes, and sequence coverage, for 10 clinical isolates from vivax malaria patients taken from two regions: Ubon Ratchathani (UBT; North-East) and Yala (YL; South)

Isolate		Total RNA (ng)	RNA integrity number (RIN)	Total read pairs^a^	Pair reads mapped to human	Percentage of reads mapped to human	Pair reads mapped to *P. vivax*	Percentage of reads mapped to *P. vivax*	Mean sequence coverage
1	UBT3086	585.2	2	29,157,609	22,771,884	78.10	346,899	1.20	1.82
2	UBT3087	392	2.4	36,549,438	29,392,764	80.42	977,753	2.68	17.67
3	UBT3089	1498	2.3	30,083,802	23,272,992	77.36	3,035,144	10.09	78.52
4	UBT3090	719.6	2.3	30,397,809	22,226,523	73.12	3,267,128	10.75	17.59
5	UBT3091	504	2.3	28,550,673	18,312,289	64.14	6,388,456	22.38	42.07
6	YL3111	627.2	2.3	29,500,938	26,039,174	88.27	580,181	1.97	3.18
7	YL3112	1005.2	2.3	32,578,915	27,732,216	85.12	913,421	2.80	4.95
8	YL3113	280	2	26,853,921	21,654,627	80.64	209,249	0.78	1.07
9	YL3114	1470	2.8	31,860,822	27,764,327	87.14	350,337	1.10	1.88
10	YL3115	641.2	2.5	32,987,343	24,102,143	73.05	3,469,628	10.52	20.13

^a^After adapter and quality trimming

### Principal component analysis

**Fig. 1:**
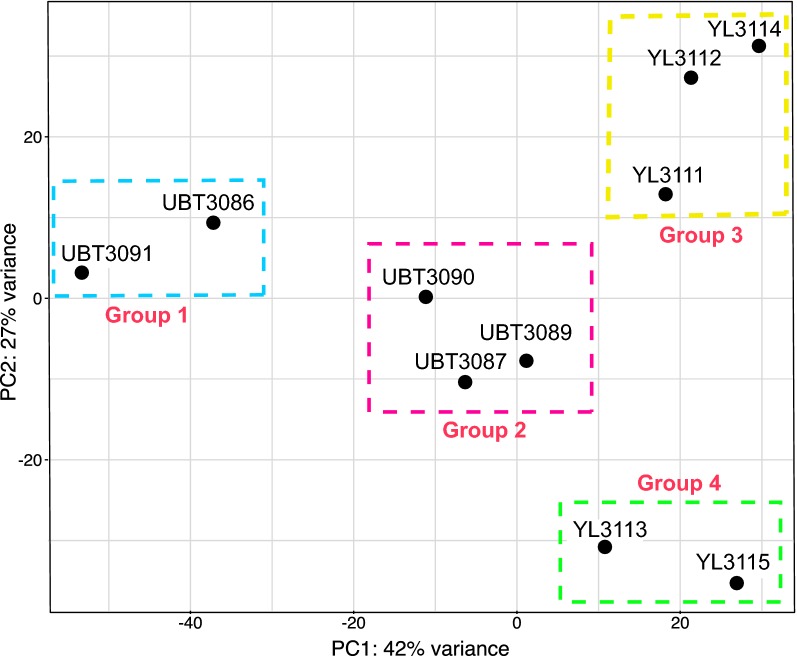
Principal component analysis (PCA) of genome-wide expression profiles for ten clinical isolates (5838 genes). The principal components were calculated from normalized read counts implemented in DESeq2. Axes represents the first and second principal components (PC1 and PC2), accounting for 42% and 27% of total variance, respectively. Each dot represents an individual isolate, and isolates were divided into four ‘patient groups’ based on the PCA

*Plasmodium vivax* infrapopulations in clinical settings usually comprise variable mixtures of different parasite life stages, which, depending on the precise compositional balance, could be reflected in stage-specific expression patterns. Unfortunately, the proportions of parasite developmental stages could not be determined from microscopic slides as these were not retained by the field hospitals. Instead, the relative proportions of parasite stages were characterized by quantifying the expression of stage-specific markers. Ten patients were separated into groups based on a principal component analysis of genome-wide transcript abundance, which was independent of age and days of patent infection (i.e. fever). The ten patients clustered into four groups using the expression profiles of 5838 genes produced by the *DESeq2* analysis (Fig. 1; Additional file 1). Together, the two largest principal components account for 69% of the total variation in transcript abundance. These groups do not reflect coverage, so YL3113 can be clearly distinguished from YL3111, YL3112 and YL3114, despite these four samples having a comparable low coverage; similarly, UBT3089 and UBT3091, which had the highest sequence coverage values do not cluster together. This reassures that the variation seen is not simply an artefact of the degree of degradation within the samples. The study focused on patient group 4 because these patients had a distinct MSP-7 expression profile (see below). Patient group 4 included two patients that had experienced longer patency (i.e. 7 days), than patients in groups 1–3, who had experienced fever for 2–3 days. These four patient groups were the basis for pairwise comparison of PvMSP-7 expression and analysis of differential gene expression. The relatedness among the ten parasite isolates was assessed to rule out the possibility that relatedness might explain the patient groups. 42,988 biallelic single nucleotide polymorphisms (SNPs) were extracted from mapping data for each RNA-seq dataset. Principal component analysis of these SNPs shows that isolates cluster by geographical origin, and do not recapitulate the patient groups (Additional file 2). Therefore, affinities among isolates based on transcript abundance are not simply a reflection of genetic relatedness or geographical origin.

### PvMSP-7 expression profiles

**Fig. 2:**
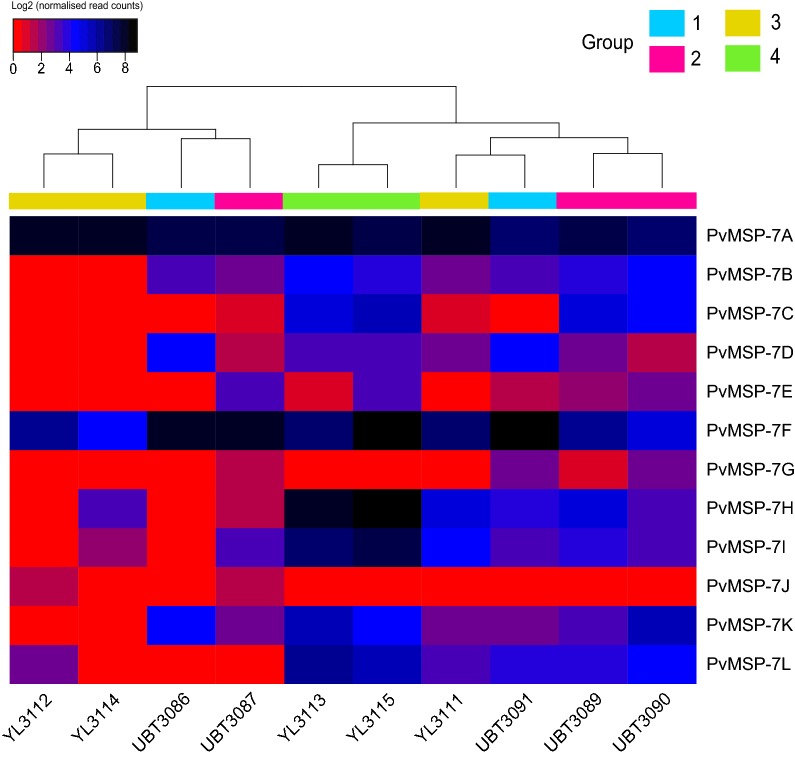
Expression profiles for 12 PvMSP-7 paralogs in ten clinical isolates. The heat-map describes log2-transformed normalized read counts from the DESeq2 analysis; shading reflects transcript abundance from low (red) to high (black) shading. Clustering in the y-axis dendrogram reflects similarity in profile between genes. Clustering in the x-axis dendrogram indicates the similarity of PvMSP-7 expression profile between patients; for instance, patient group 4 isolates display a very similar profile for PvMSP-7

To investigate the expression of PvMSP-7 during diverse clinical infections, and to examine variation in transcriptional profile within the family, normalized transcript abundance in each isolate for the 12 paralogous genes was plotted as a heat map, shown in Fig. 2. From the relationships among isolates, seen across the top of Fig. 2, it is immediately clear that there is substantial variation in PvMSP-7 expression among patients, which only partly coincides with patient groups, i.e. genome-wide expression profile. There are also consistent differences among PvMSP-7 paralogs; 7A and 7F display higher abundance than all others (mean log2 normalized read counts of 7.76, 7.19, and 6.28, respectively). Even in patients with otherwise low PvMSP-7 expression, such as YL3112 and YL3114, these two paralogs maintain the same high level of expression. Conversely, 7E, 7G, and 7J were uniformly rare transcripts (mean log2 normalized read counts of 1.44, 0.81 and 0.33, respectively); the latter is perhaps unsurprising since PvMSP-7J is a pseudogene [13]. Other paralogs are only transiently abundant, for example, 7H and 7I (mean log2 CPM of 4.03 and 3.58 respectively), were highly abundant in patient group 4 only (mean log2 CPM of 8.65 and 7.29 respectively); indeed, in these two patients that had experienced longer patency, their expression was comparable to 7A and 7F. Hence, when differential expression analysis was performed on normalized transcript abundance values (data shown in Additional file 3), significant differences in the abundance of PvMSP-7H were found (mean log2FC = 4.86; mean p_adj_ = 0.026) when patient group 4 was compared with all other groups; and likewise for 7I (mean log2FC = 3.84; mean p_adj_ = 0.024) when patient group 4 was compared with groups 2 and 3, and 7C (log2FC = 6.48; p_adj_ = 0.0002), when compared with patient groups 1 and 3. Finally, MSP-7L (log2FC = 3.64; p_adj_ = 0.007), 7K (log2FC = 4.54; p_adj_ = 0.03) were differentially expressed when patient group 4 was compared with group 3 only. In contrast, no significant differences in PvMSP-7 expression were found in pairwise comparisons among patient groups 1–3.

### Differential gene expression analysis

**Fig. 3:**
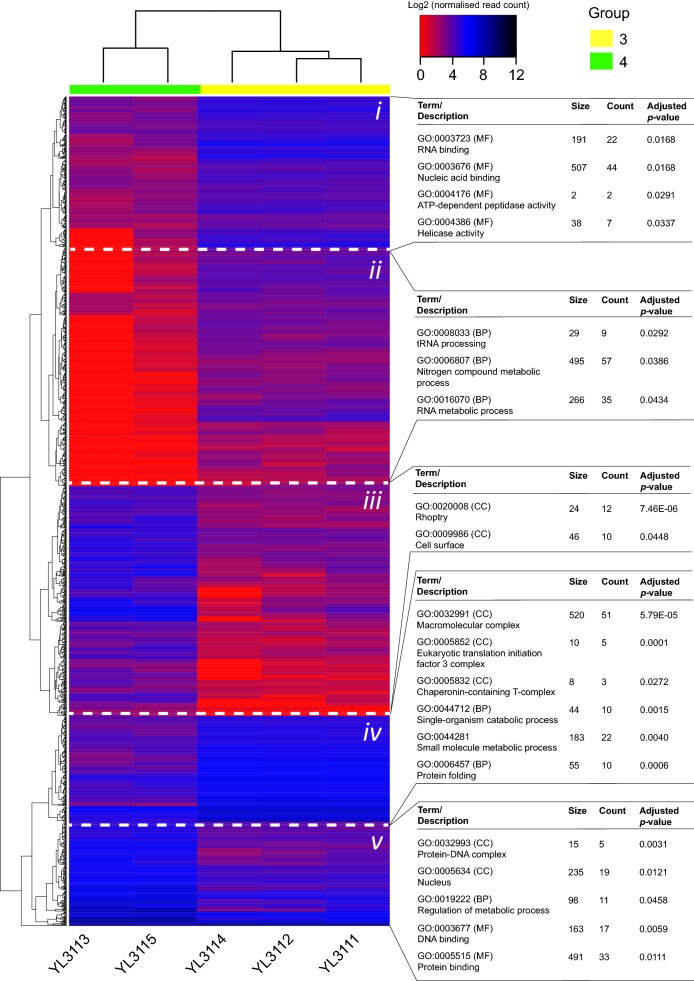
Expression profiles of differentially expressed genes (DEGs) in a comparison of patient groups 3 and 4 (n = 1493). The heat-map shows expression profile across five clinical isolates, generated from log2-transformed normalized read counts. Clustering in the y-axis dendrogram reflects similarity in profile between DEGs. Clustering in the x-axis dendrogram indicates the similarity of differential expression between patients. The heat-map is divided into five sectors based on y-axis clustering. For each sector, the results of enrichment analysis of Gene Ontology (GO) terms are shown on the right. GO terms with significant enrichment within a given heat-map sector are listed with their description, ontological category [i.e. biological process (BP), molecular function (MF), cellular compartment (CC)], frequency in the background gene set (‘size’) and incidence within the heat-map sector (‘count’). Significance threshold was p-value < 0.05 after Bonferroni correction

Given that critical differences were seen in expression profile among PvMSP-7 paralogs in distinct patient groups, it was necessary to better understand the global gene expression landscape in these groups, to help explain the PvMSP-7 patterns. Having identified differentially expressed genes (DEGs) in pairwise comparisons of patient groups using DESeq2, the log2 normalized read counts for all DEGs in each comparison were plotted in heat-maps. The highest number of DEGs (1493; Additional file 3) was observed between patient groups 3 and 4, and these are plotted in Fig. 3. The heat-map relates gene expression profiles (rows) with clinical isolates (columns); the vertical dendrogram clusters genes based on their expression profile, the horizontal dendrogram clusters isolates based on their global expression profile. The heat-map is divided into sectors based on clades observed in vertical dendrogram, and Gene Ontology enrichment analysis was performed on each sector.

The 1493 DEGs identified between patient groups 3 and 4 may be segregated into five sectors (*i* to *v*). Genes in sector *i* (*n *= 252) and *ii* (*n *= 456) were significantly less abundant in group 4 and enrichment analysis showed that these genes are associated with *Nucleic acid binding*, *ATP*-*dependent peptidase activity*, *helicase activity*, *tRNA processing*, *nitrogen compound metabolism*, and *RNA metabolism*. Genes in sector *iv* (*n *= 192) were also more abundant in group 3 and GO terms associated with these related to *macromolecular complex*, *eukaryotic translation initiation factor 3 complex*, *Chaperonin*-*containing T*-*complex*, *small molecule metabolic process*, and protein folding. Conversely, genes in sector *iii* (*n *= 403) and *iv* (*n *= 190) were significantly more abundant in group 4 isolates. These genes were enriched for GO terms associated with *Rhoptry*, *cell surface*, *protein*–*DNA complex*, *nucleus*, *regulation of metabolic process*, *DNA binding*, and *protein binding*. PvMSP-7K, 7I, 7H, 7C, and 7L were included among the sector *iii* genes.

Comparisons of patient group 4 with 1 and 2 identified fewer DEGs but consistent patterns of differential expression. A heat-map of the 251 DEGs detected between Groups 4 and 1 is shown in Additional file 4. Sector *i* contains genes that are significantly less abundant in group 4 (*n *= 125); these are associated with GO terms for *Cell surface*, *Maurer’s cleft, host cell surface binding*, and *Uridylyltransferase activity*, and implicate such genes as reticulocyte binding surface protein (PvP01_00004240; log_2_ FC = 9.27, p_adj_ = 2.97e^−15^), tryptophan-rich protein (PvP01_0504200; log_2_ FC = 7.32, p_adj_ = 0.001) and a small heat shock protein HSP20 (PvP01_0518800; log_2_ FC = 5.17, p_adj_ = 0.034). Sector *ii* also contains genes that are expressed more in group 1 isolates (*n *= 71); here, GO terms are associated with *crystalloid*, *cell surface*, and *host cell cytoplasm*. Sector *iii* (*n *= 22) and sector *iv* (*n *= 33) contain genes that were more abundant in group 4 isolates. GO terms enriched within this gene set concern *Rhoptry, cell surface*, and *proteolysis*, and implicate known invasion-related genes such as high molecular weight rhoptry protein 3 (PvP01_0703800; log_2_ FC = 6.38, p_adj_ = 0.006), high molecular weight rhoptry protein 2 (PvP01_0727900; log_2_ FC = 5.81, p_adj_ = 0.0004), 6-cysteine protein (PvP01_1136400; log_2_ FC = 6.16, p_adj_ = 0.008) and merozoite surface protein MSA180 (PvP01_0814200; log_2_ FC = 7.25, p_adj_ = 9.46e^−5^). The comparison of patient groups 2 and 4 is shown in Additional file 5. 351 DEGs were identified and these segregate into five sectors in the heat-map. No distinct expression patterns are observed within sector *i* (*n *= 99) and *ii* (*n *= 49). Genes in sector *iii* (*n *= 47) and *iv* (*n *= 142) were less abundant in group 4 isolates. They are associated with functional terms for *purine metabolism* and *host cell surface binding*.

While the analysis of differential expression shows that these clinical isolates have different transcriptional landscapes, and clearly associate expression of PvMSP-7C, 7H and 7I with patient group 4 only, relatively few DEGs contribute to the enrichment analysis and so enriched functional terms have limited capacity to explain the transcriptional differences. Among the DEGs, various specific markers of parasite development were observed, such as schizont egress antigen-1 (SEA1; PVP01_0607000), which was more abundant in patient group 4 than 3 (log_2_ FC = 4.92; p_adj_ = 9e^−06^) and gametocyte development protein 1 (GDV1; PVP01_0734100), which was most abundant in patient group 3 than 4 (log_2_ FC = 3.73; p_adj_ = 0.0001). Thus, to explore developmental composition as an explanation of transcriptional differences, associations between PvMSP-7 expression profiles and such specific markers were sought using co-expression analysis.

### Co-expression analysis

Co-expression analysis using the *coseq* package was applied to integrate DEG transcript abundance in patient group 4 with each of the other groups. Co-expression analysis would typically be applied to a series of longitudinal samples taken over a time-course, but here it was applied it to ten clinical isolates after ordering these by patient group. Given that patient group 4 has a significantly different MSP-7 expression profile to other groups, the analysis examined whether these genes are co-expressed in group 4 with developmental stage-specific markers. If so, associations between MSP-7 and developmental markers would help to explain the essential differences between the patient groups.

**Fig. 4:**
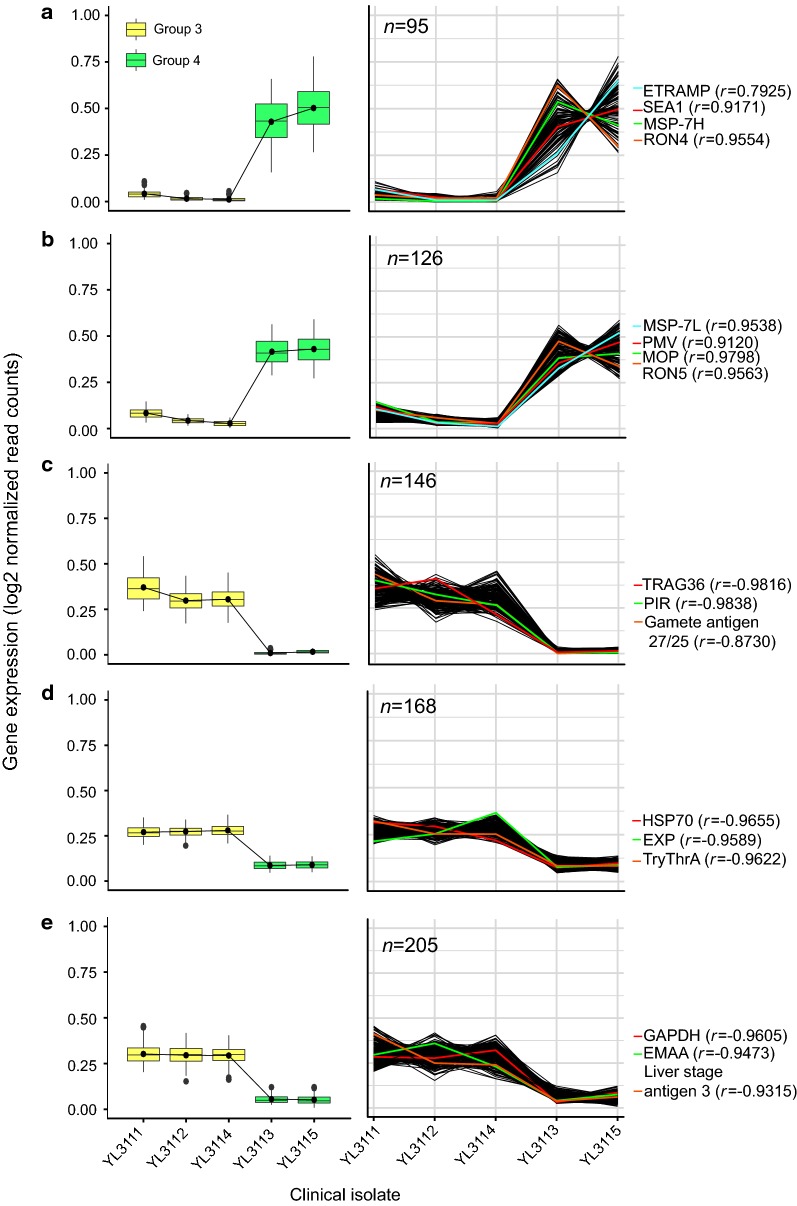
Co-expression analysis of genes differentially expressed between patient groups 3 and 4. *Coseq* was used to generate clusters of genes with positively correlated transcription profiles. 1493 DEGs formed 14 clusters; five clusters with dynamics that clearly distinguish the two patient groups are shown here. **a**, **b** Concern transcripts that are abundant in patient group 4 and positively correlated with PvMSP-7 expression. **c**–**e** Concern transcripts that were abundant in patient group 3, and were negatively correlated with PvMSP-7l. Boxplots (left) represent expression values for all genes in a cluster (Log2-transformed, normalized read counts) from an individual patient, shaded by patient group; the black line connecting boxplots is the mean expression value of all genes in the cluster. **a** PvMSP-7K, 7I, 7H and 7C (only 7H is shown) were co-expressed with 91 other genes including various invasion-related genes such as ETRAMP, SEA1, and RON4. **b** A fifth MSP-7 gene, PvMSP-7L, was co-expressed with 125 other genes including PMV, MOP, and RON5. **c** A gametocyte stage-specific marker, gamete antigen 27/25, was absent in patient group 4 but positively correlated with TRAG36 and PIR genes. **d** A cohort of 168 genes that are abundant in patient group 3 but absent in group 4. **e** Another stage-specific marker, liver stage antigen 3, is abundant in patient group 3 and co-expressed with 204 other genes. Pearson’s correlation coefficient (r) for abundance values of MSP-7H and selected genes are shown

The correlation of expression profiles for 1493 DEGs across patient groups 4 and 3 produced 14 transcript clusters (Additional file 6). Of these, five clusters (including 740 DEGs) are of interest because they describe a dynamic that clearly distinguishes the patient groups; these are shown in Fig. 4. The top panel (Fig. 4a) describes a cluster that is more abundant in patient group 4 than group 3. This cluster included PvMSP-7K, 7I, 7H and 7C, reflecting the differential expression analysis, in which log_2_ fold change ranged from 4.21 to 6.48. These MSP-7 transcripts were co-expressed with several stage-specific genes with known functions in erythrocyte invasion, such as early-transcribed membrane protein (ETRAMP, PvP01_0618300), schizont egress antigen-1 (SEA1, PvP01_0607000), rhoptry neck protein 4 (RON4, PvP01_0916600). Figure 4b describes a transcript cluster that included MSP-7L (log_2_ FC = 3.64) and other invasion-related genes, such as plasmepsin V (PMV, PvP01_1231100), merozoite organizing protein (MOP, PvP01_0715400), and rhoptry neck protein 5 (RON5, PvP01_0517600).

Contrasting with these two clusters, Fig. 4c–e displays transcriptional profiles that are most abundant in patient group 3, declining in group 4. These clusters include other stage-specific markers indicative of different parasite life stages, such as gamete antigen 27/25 (PvP01_0422700), sporozoite and liver stage tryptophan-rich protein (TryThrA, PvP01_0532600), and liver specific protein 3 (PvP01_0405000). Besides these, various multi-copy gene families typical of asexual-stage cells are co-expressed, such as tryptophan-rich protein (TRAG36, PvP01_0119200), *Plasmodium* interspersed repeat (PIR, PvP01_0816000), *Plasmodium* exported protein (EXP, PvP01_0300700) and erythrocyte membrane-associated antigen (EMAA, PvP01_0103700). Pearson’s correlation coefficient (*r*) was estimated for each stage-specific marker in relation to MSP-7H. Overall, positive correlations (*r *> 0.90) were observed for schizont/merozoite-stage expression gene while, negative correlations (*r *> − 0.85) were seen with sporozoite, liver, and gametocyte stage markers. MSP-7I and 7C genes enriched in patient group 4 gave consistent results when correlated with stage-specific genes.

The correlation of expression profiles for 251 DEGs across patient groups 4 and 1 produced seven transcript clusters (Additional file 6); four are shown (including 208 DEGs) in Additional file 7. In this comparison, PvMSP-7H and 7C were significantly differentially expressed (log_2_ FC = 5.31, 7C and 6.33, respectively). These two PvMSP-7 paralogs were placed in different clusters (Additional file 7a and b), although weakly positively co-expressed (*r *> 0.60). They were significantly co-expressed with various invasion-related genes, including PIESP1, PMV, rhoptry neck protein 3 (RON3, PvP01_1469200), serine-repeat antigen-1 (SERA, PvP01_0417100), subtilisin-like protease 3 (SUB3, PvP01_1026800), and high molecular weight rhoptry protein 3 (RhopH3, PvP01_0703800). Genes negatively associated with PvMSP-7H and 7C included two gametocyte stage-specific markers (gamete release protein (PvP01_0115300) and gamete antigen 27/25 (PVP01_0422700).

The correlation of expression profiles for 351 DEGs across patient groups 4 and 2 produced seven transcript clusters (Additional file 6); four clusters (comprising 176 DEGs) are shown in Additional file 8. These clusters present a similar picture to that above. Expression of MSP-7H and 7I was positively correlated with SEA1 (Additional file 8b), but contrasted with expression of liver specific protein 3 (Additional file 8c), and gamete antigen 27/25 (Additional file 8d), as well as various *Plasmodium* exported proteins and tryptophan-rich proteins associated with asexual stages.

From the differential expression and co-expression analyses, it is apparent that MSP-7H and 7I are positively co-expressed with the schizont stage-specific marker SEA1, and various other invasion-related genes. This indicates that isolates from patient group 4 are distinguished from other groups by a greater proportion of schizonts in the parasite infrapopulation, relative to other life stages. Accordingly, MSP-7H and 7I have a negative association with liver-stage and gametocyte-stage markers.

### Peptide microarray assay of PvMSP-7 linear B-cell epitopes

**Fig. 5:**
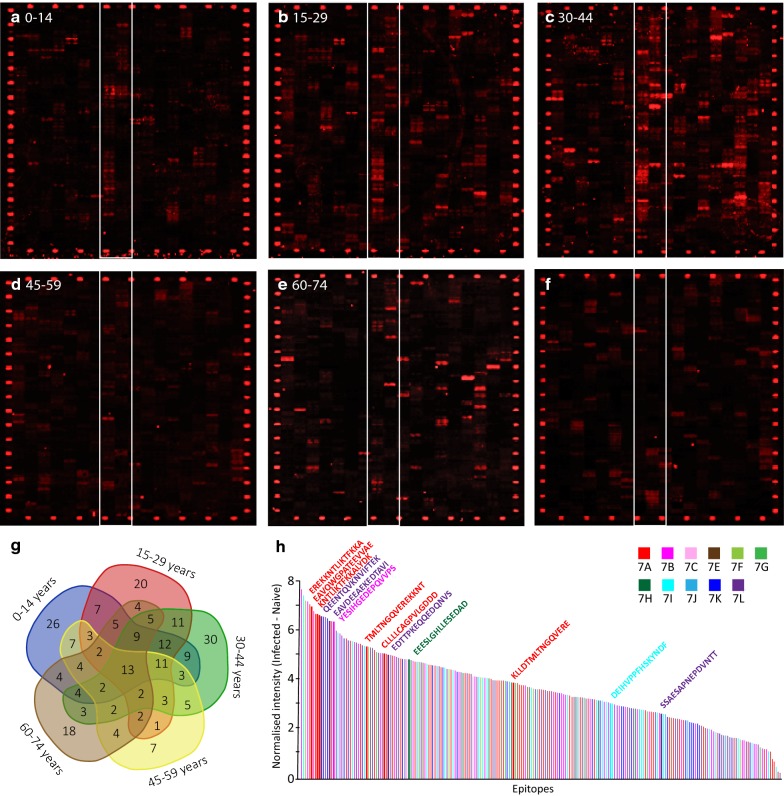
Mapping of putative B-cell epitopes across 12 PvMSP-7 paralogs by peptide microarray. Plasma from naturally infected vivax malaria patients was applied to a custom peptide microarray to determine linear B-cell epitopes. The microarray consisted of 2346 spots, representing 1173 peptides (each 13 amino acids) derived from the predicted protein sequences of 12 PvMSP-7 paralogs, overlapping by one amino acid and spotted in duplicate. **a**–**e** Fluorescent response following incubation of the peptide microarray with patient plasma, pooled by age in years: 0–14 (**a**), 15–29 (**b**), 30–44 (**c**), 45–59 (**d**), 60–74 (**e**), and application of a Cy3 (red) conjugated, secondary goat anti-human IgG antibody. Each spot on the peptide microarray corresponds to one peptide. **f** Negative control consisting of plasma derived from naïve patients living in malaria-free areas. Control peptides (HA) are located around the edge of the array and are stained with Cy3 (red) conjugated anti-HA antibody. For comparison, the strongest responding spots are highlighted between the white lines. **g** A Venn diagram showing the overlap in predicted linear B-cell epitopes across five patient age groups. 13 peptides that gave significant responses in all experimental groups were observed to be present in all age groups. **h** Peptides that gave fluorescent responses significantly greater than the naïve control in one or more age groups (N = 236) are presented in order of response intensity and shaded by PvMSP-7 paralog. The predicted amino acid sequence of the epitope is given for 13 peptides that were significant in all five age groups

Immuno-reactive linear B-cell epitopes were identified by screening plasma from naturally-infected individuals with a customized PvMSP-7 peptide microarray, which consisted of 1173 15-mer peptides spanning the complete coding sequence of 12 PvMSP-7 paralogs (see Additional file 9). The peptide microarray was incubated with plasma from patients with single *P. vivax* infection, pooled into five age groups. The purpose of separating plasma into these arbitrary age divisions was not to examine the effect of age per se, but to ensure that the epitopes identified were significantly more responsive than naïve samples regardless of patient age. A secondary goat anti-human IgG antibody with conjugated fluorophore (DyLight 680) was applied to determine the plasma reactivity profile for each peptide. Figure 5 shows that plasma from the 30–44 age group displayed immuno-reactivity to the most epitopes (Fig. 5c), while the fewest responses was observed from the 45–59 age-group (Fig. 5d). A negative control comprised plasma from malaria-naïve individuals; some fluorescence was observed from the negative control, but these responses were relatively weak. Furthermore, responses from the naïve control did not generally coincide with immune-reactive peptides bound by plasma samples. By comparing the spots between the white lines in Fig. 5a–f, the characteristic response seen in infected plasma (Fig. 5a–e) is not reproduced in the naïve control (Fig. 5f).

Fluorescent intensity values were corrected for background intensity (i.e. non-specific binding by secondary antibody), and then also for inter- and intra-array variation (Additional file 10). Normalized intensity values were then compared to corresponding responses from the naïve control using the LIMMA package [47] to determine the significance of each response. The number of significantly immunogenic peptides found among 12 PvMSP-7 paralogs was 238, but these belonged disproportionately to specific paralogs and specific plasma age groups. Figure 5h arranges these putative B-cell epitopes in order of decreasing spot intensity. Four PvMSP-7 paralogs [PvMSP-7A (21%), 7B (18%), 7K (12%) and 7L (20%)] contained a large proportion of predicted epitopes; six other PvMSP-7 proteins (PvMSP-7C, 7E, 7F, 7G, 7H, and 7J) each contributed less than 5% of putative epitopes, while no significant responses were detected against PvMSP-7D.

Figure 5g shows the number of putative epitopes by plasma age group as a Venn diagram. Age group of 30–44 displayed the highest number of the significant intensity values (n = 123), followed by 0–14 (n = 121), 15–29 (n = 110), 60–74 (n = 83), and 45–59 (n = 71). The responses for the 45–59 age cohort are weak by comparison with others, and not, on appearance, substantially different to the naïve cohort. The lack of response from the 45–59 age cohort cannot be explained by sampling artefact, given that it consisted of 10 individuals sampled at different times. Nevertheless, although plasma from the 45–59 age cohort are not generally more responsive than the naïve plasma, all of the ‘universal’ epitopes identified below were specifically recognized by these infected plasma, and to a degree significantly greater than naive plasma.

**Fig. 6:**
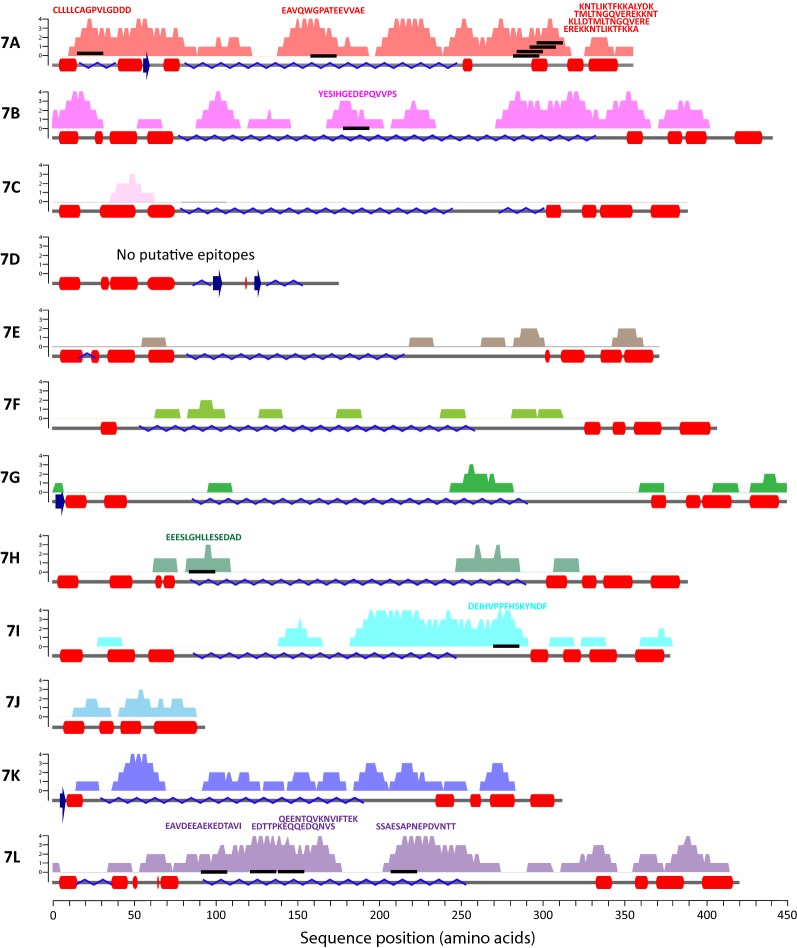
Observed linear B-cell epitopes mapped to predicted secondary protein structures of 12 PvMSP-7 proteins. The cartoon depicts the intensity and position of linear B-cell epitopes identified by microarray within each PvMSP-7 protein. The x-axis shows the secondary structure of each paralog to scale, as predicted by JPred4 [65]: coiled-coil (grey line), alpha-helix (red lozenge), beta-strand (blue arrows). The intrinsically unstructured/disordered regions (blue zig-zag line) was predicted using the GeneSilico MetaDisorder service [66]. The positions of 236 peptides giving significant responses from the microarray are plotted; the y-axis measures the epitope coverage of a given amino acid position (maximum of four epitopes). The positions of 13 ‘universal’ epitopes observed in all assays (see Fig. 5g) are marked by black bars and their corresponding epitope sequences

Of the 236 spots that responded significantly to infected plasma, 13 putative epitopes were detected in all age groups (Table 3). These universal epitopes were found in five PvMSP-7 proteins (PvMSP-7A, 7B, 7H, 7I, and 7L), but 6/13 were derived from PvMSP-7A. These universal epitopes are not always among the most immuno-reactive (Fig. 5h), but 6/13 occur among the 10% most responsive peptides and three of these are derived from MSP-7A. The position of these putative epitopes in relation to predicted protein secondary structure is shown in Fig. 6.

**Table 3:** Sequence, parent gene and structural position of 13 PvMSP-7 peptides that gave significant responses on the peptide microarray in all age groups, compared to the negative control

Epitope sequence	PvMSP7 paralog	Position within protein sequence (aa)	Signal intensity		p_adj_^a^
			Infected^a^	Naive	
CLLLLCAGPVLGDDD	A	10–25	10.0182341	6.403	0.003
EAVQWGPATEEVVAE	A	158–173	10.9368279	7.083	0.002
KLLDTMLTNGQVERE	A	298–313	9.47392489	6.693	0.009
TMLTNGQVEREKKNT	A	302–317	9.09588506	5.900	0.014
EREKKNTLIKTFKKA	A	310–325	8.31646604	4.383	0.004
KNTLIKTFKKALYDK	A	314–329	5.52137066	0.000	0.012
YESIHGEDEPQVVPS	B	178–193	8.86022031	5.274	0.009
EEESLGHLLESEDAD	H	83–98	9.86610447	6.932	0.011
DEIHVPPFHSKYNDF	I	272–287	9.52211097	7.151	0.016
EDTTPKEQQEDQNVS	L	91–106	8.74109552	5.510	0.012
QEENTQVKNVIFTEK	L	123–138	9.29166832	4.963	0.010
EAVDEEAEKEDTAVI	L	139–154	11.8856457	7.626	0.002
SSAESAPNEPDVNTT	L	207–222	10.2973557	8.476	0.026

^a^Mean value across all age groups

## Discussion

Ten patients infected with vivax malaria were recruited from two areas of Thailand and examined the expression of, and immune response to, MSP-7 paralogs. Analysis of gene expression in asynchronous *P. vivax* infrapopulations is uncommon, but a recent study by Kim et al. [50] generated transcriptomes for three Cambodian clinical isolates; their parasite gene expression profiles had strong positive correlations (r^2^ > 0.8), regardless of the differences in the proportions of developmental stages in the original infections. The current analysis presents another picture; isolates display diverse parasite expression profiles that appear to reflect differences in the developmental composition of parasite infrapopulations. Variation in expression profile between PvMSP-7 paralogs in the clinical isolates has been identified that correlates with broader differences in developmental stage-specific gene expression, as well as with substantial variation in the natural immune response to PvMSP-7 proteins; together, these findings indicate that gene paralogy is an important factor in MSP-7 vaccine design.

MSP-7 is characterized by its developmental regulation during the merozoite stage, its cell-surface localization, and it is a prominent role in the cell invasion machinery [11, 12, 16, 18, 19, 24, 51–53]. So the positive co-expression of PvMSP-7C, 7H and 7I with a schizont stage-specific marker (i.e. SEA1), as well as a cluster of other invasion-related genes, in patients that had experienced longer patency is consistent with the current understanding. These MSP-7 genes are part of an invasion-related gene cohort, transcription of which is known to be specifically upregulated during schizogony through the activation of RNA polymerase II, in contrast to the majority of blood-stage genes, which are constitutively expressed throughout the IDC [54]. Based on the abundance of specific markers for schizont, liver and gametocyte stages, it is clear that the composition of parasite infrapopulations in the ten patients varied, and that these differences caused the result seen in Fig. 1. Contrasting with these conventional observations, the high abundance of PvMSP-7A and 7F in all patients and, by extension, their lack of correlation with stage-specific markers, indicates that these two paralogs are expression throughout the IDC. Thus, the authors contend that PvMSP-7A and 7F have undetermined roles during ring and trophozoite stages in addition to the familiar function on the merozoite surface. PvMSP-7A and 7F show significantly less structural variation in natural populations than other family members and higher rates of purifying selection, which offers circumstantial evidence for their being functionally differentiated [12, 21]. It can only speculated as to what these additional functions might be. PvMSP-7 was seen to be a ligand for the host receptor P-selectin [55]. This study did not determine which paralog, if not all, was implicated in this interaction, but this makes the general point that binding specificities for host proteins may vary widely within the gene family, and those of PvMSP-7A and 7F may relate to intracellular proteins encountered by asexual blood stages, in addition to host cell surface proteins bound by invading merozoites.

**Fig. 7:**
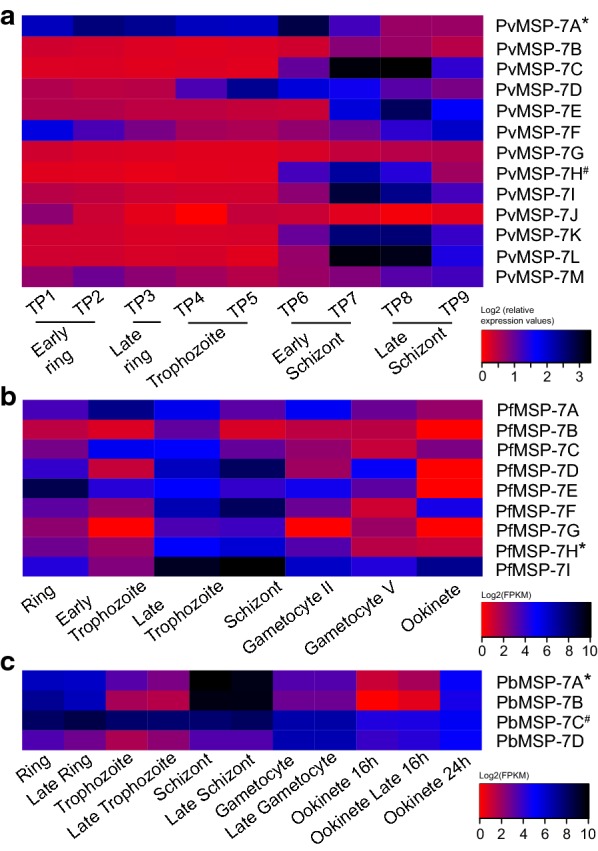
Comparison of MSP-7 expression profiles from published transcriptomes of synchronized cell cultures in multiple *Plasmodium* species. **a** Expression profiles for 12 PvMSP-7 genes in *P. vivax*, as determined by microarray analysis [25]. **b** Expression profiles for seven PfMSP-7 genes in *P. falciparum* across seven blood-stages, as determined by cDNA sequencing [56]. **c** Expression profiles for four PbMSP-7 genes in *P. berghei*, across five life stages, as determined by RNA-seq [57]. All values are log2-transformed. Shading represents transcript abundance from low (red) to high (black). An asterisk and hash indicate two sets of orthologous genes in different species respectively

If this view that variation in expression reflects functional diversity is correct, then the same consistent patterns should be evident in transcriptomes from synchronous cell cultures. Figure 7 describes the MSP-7 expression profiles in synchronous cell cultures of *P. vivax* [25], *P. falciparum* [56], and *P. berghei* [57]. None of these studies explicitly examined MSP-7; note that the species differ in the number of paralogs. The *P. vivax* transcriptome was described by Bozdech et al. [25] who used microarrays to capture transcriptional changes during the IDC over 48 h, from the early ring to schizont stage. 11/13 PvMSP-7 genes increased expression from early schizont stage to late schizont stage. Consistent with the present data, PvMSP-7A and 7F were expressed most strongly throughout the IDC, while MSP-7G and 7J were the rarest forms (Fig. 7a; [25]). López-Barragán et al. [56] used RNA-seq to measure gene expression in synchronized *P. falciparum* cultures, from ring- to ookinete stage. Their data present a similar pattern to the situation in *P. vivax*; two paralogs, PfMSP-7A and 7I were highly abundant in all stages, while PfMSP-7G and 7H only become abundant in the schizont (Fig. 7b; [56]). Finally, a *P. berghei* transcriptome based on RNA-seq from synchronized ring to ookinete stages [57] also shows that one paralog (PbMSP-7C) maintains high abundance throughout, while other paralogs (PbMSP-7A and 7B) peaked in expression during the schizont stage (Fig. 7c; [57]). Clearly, in all species, MSP-7 expression peaks generally during the late trophozoite-schizont transition, when merozoites are forming. However, in all species, there is a minority of paralogs that maintain abundant expression throughout the IDC, further supporting the view that the MSP-7 family may have additional functions besides their established role on the surface of the invading merozoite.

The presence of these constitutively expressed paralogs (within the context of the IDC) in multiple species raises the possibility that they are orthologous, and thus, suggestive of functional differentiation that is conserved across the genus. There are two PvMSP-7 genes with unambiguous orthologs in the other species: 7A and 7K [13]. PvMSP-7A (orthologous to MSP-7H in *P. falciparum* and MSP-7A in *P. berghei*) did not show similar expression patterns across the IDC. While PvMSP-7A expression began in the early ring stage and peaked at early schizont stage [25], PfMSP-7H was transcribed on from late trophozoite to gametocyte II, and absent after gametocyte stage V [56]. PbMSP-7A likewise did not maintain its expression throughout the IDC, but was strongly associated with the schizont stage [57]. Meanwhile, PvMSP-7K (orthologous to MSP-7C in *P. berghei*) is expressed exclusively during early to late schizont stage [25] while, PbMSP-7C was constitutively expressed throughout the IDC [57]. It is clear from the MSP-7 gene phylogeny [13] that the constitutively expressed paralogs in each species do not cluster together as orthologs, and are, in fact, drawn from different lineages within the family. The combination of longer-expressed and briefly-expressed genes is conserved but the genes performing these contrasting roles are not. This suggests that the functional need for different roles is constant but is fulfilled in different species by a rapidly changing MSP-7 repertoire.

PvMSP-7A has been identified, from among the several paralogs, as eliciting the strongest and most consistent immune responses in natural vivax malaria infections. This may be because PvMSP-A is more exposed to the immune system, being more abundant than other paralogs that are expressed by a smaller proportion of the parasite infrapopulation. Peptide arrays have been used successfully elsewhere to map linear B-cell epitopes of various vaccine candidates, including other merozoite surface proteins such as MSP1 and MSP3 [58]. Jaenisch et al. [59] used peptide microarrays assayed with serum from malaria patients to test a range of *P. falciparum* proteins preselected for their in silico antigenicity. Among the significant epitopes they discovered were several specific to PfMSP7. Peptide arrays have also been used to examine variant antigens, which are certainly important mediators of the immune response, but more challenging to study than low-copy number proteins, and to identify epitopes of RIFIN and STEVOR proteins that are associated with severe falciparum malaria in Malian children [60].

These studies have their limitations; the peptide arrays are printed using bacterial cell systems that may not accurately recreate eukaryotic protein decorations crucial to recognition in nature. They also do not address conformational or discontinuous epitopes. Nonetheless, antibody responses to malaria antigens on these arrays correlate well with measurements of purified proteins using enzyme-linked immunosorbent assays (ELISAs) [58]. In the present study, only epitopes that were significant in all age cohorts were selected, yet responses from the 45–59 age cohort were clearly lower than other groups, perhaps limiting the number of universal, significant epitopes that could be detected.

Clearly, the natural immune responses to PvMSP-7 would be expected to lead to some level of protection against infection. However, associations with protection are inconsistent, probably reflecting the complexity of each local situation and their resident parasite populations. Crompton et al. [58] found that high antibody titres to several vaccine candidate antigens (LSA-3, MSP-1, and MSP-2) had no statistically significant association with protection from uncomplicated malaria in Malian children. In contrast, the same markers were predictive of protection in Kenyan children [61]. Finding a somewhat intermediate position, Nixon et al. [30] examined the relationship between epitope-specific PfSEA-1 antibody levels and protection from *P. falciparum* in a longitudinal treatment-reinfection cohort in Kenya. They observed that antibodies to three epitopes were associated with 16 to 17% decreased parasitaemia. The MSP-7 epitopes identified here would likely meet a similar situation, identifying the relevant B-cell epitopes enables further studies of the protective properties of PvMSP-A but, in all likelihood, these natural responses will need to be enhanced through optimization of adjuvant and delivery protocols if they are to protect against malaria.

## Conclusion

With these results, the impact that functional variation within the PvMSP-7 gene family could have for vaccine development can be assessed. MSP-7 proteins are promising vaccine candidates due to their cell surface position and their prominence in interactions between host and invading parasite [14]. In this, they resemble the reticulocyte-binding protein (RBP) family, another multi-copy gene family developed as vaccine candidates in both *P. vivax* [62] and *P. falciparum* [63, 64]. Careful consideration has been given to diversity within the RBP family and to the optimal selection on gene copies. Of the eleven PvRBP genes, two paralogs, PvRBP1a and PvRBP1b, are both favoured as vaccine candidates as they localize to the microneme during schizogony and bind host ligands during reticulocyte invasion [62]. Han et al. favoured these two paralogs as vaccine candidates because they displayed a consistent antigenicity that elicited a protective immune response in a mice model. PvMSP-7 antigens vary widely, but consistently, in their expression during blood infections, and in their antigenicity with respect to B-cell responses. This study has shown that, as with PvRBP, it cannot be assumed that the 12 MSP-7 paralogs are equal in their antigenic properties, or equally plausible as vaccine candidates. Previous work has shown that MSP-7A is among the most structural conserved of PvMSP-7 paralogs in natural *P. vivax* populations [20–22]; this work has shown that PvMSP-7A is also expressed for the longest interval during blood infections and is the most immunogenic antigen. Hence, of all PvMSP-7 gene copies, PvMSP-7A is the optimal selection for vaccine development.

## Additional files

**Additional file 1.** Transcript abundance values (CPM) for RNA-seq data mapped to 5838 *Plasmodium vivax* PvPO1 genes. Log2 transformed normalized read counts for 5838 genes generated by *DEseq2* after mapping RNA-seq data with *Tophat2*.

**Additional file 2.** Principal component analysis (PCA) of ten clinical isolates based on transcriptome-wide single nucleotide polymorphisms (SNPs). RNA-seq data for each clinical isolate was mapped to the *P. vivax* PvPO1 reference genome (5838 genes) and SNPs (N = 42,988) were extracted using the Genome Analysis Toolkit, version 3.7. SNPs were filtered using the linkage disequilibrium pruning method and PLINK version 1.9. A sliding window size of 50 was set across the transcriptome, advancing with steps of five SNPs, and SNPs with a threshold value above 0.5 were removed. Isolate identity and Patient Groups are labelled. Geographical origin is denoted by coloured symbols: red circle (Yala), blue square (Ubon Ratchathani).

**Additional file 3.** Genes showing significant differential expression in pairwise comparisons of Patient Groups. Results of differential expression tests in pairwise comparisons of Patient Groups 1–4 using DEseq2. Fold-change is log2-transformed. p-value is Bonferroni-corrected for multiple tests. Differentially expressed genes are defined as having an adjusted p value < 0.05 and are shaded red. Significant MSP-7 genes are listed first.Genes showing significant differential expression in pairwise comparisons of Patient Groups. Results of differential expression tests in pairwise comparisons of Patient Groups 1–4 using DEseq2. Fold-change is log2-transformed. p-value is Bonferroni-corrected for multiple tests. Differentially expressed genes are defined as having an adjusted p value < 0.05 and are shaded red. Significant MSP-7 genes are listed first.

**Additional file 4.** A heatmap describing the profiles of 251 differentially expressed genes (y-axis) between Patient Groups 1 and 4 (x-axis). Data displayed are log2 transformation of normalized raw reads using DESeq2. The heatmap is divided into four sectors. Significant GO terms and/or KEGG pathways in each sector are shown in tables on the right. GO terms and/or KEGG pathways with adjusted p-value<0.05 were deemed significant. The colour scale represents the expression level of DEGs: red (lower expression), black (higher expression). Patient Groups are labelled by colour beneath the dendrogram.

**Additional file 5.** A heatmap describing the profiles of 351 differentially expressed genes (y-axis) between Patient Groups 2 and 4 (x-axis). Data displayed are log2 transformation of normalized raw reads using DESeq2. The heatmap is divided into four sectors. Significant GO terms and/or KEGG pathways in each sector are shown in tables on the right. GO terms and/or KEGG pathways with adjusted p-value < 0.05 were deemed significant. The colour scale represents the expression level of DEGs: red (lower expression), black (higher expression). Patient Groups are labelled by colour beneath the dendrogram.

**Additional file 6.** Clusters of co-expressed genes generated from transcript abundance data and their enriched GO/KEGG terms. In pairwise comparisons of Patient Groups, transcript abundance values for DEGs were analysed with *coseq*, to produce clusters of genes with expression profiles displaying significant positive correlations. Comparison of Patient Groups 3 and 4, 2 and 4 and 1 and 4 generated 14, 7 and 6 clusters respectively; the membership of these clusters is shown in three separate tabs, with notable stage-specific genes and MSP-7 shaded red. Also shown are the results of enrichment analyses for selected clusters in each comparison (i.e. those displaying a distinct profile with respect to patient group 4), and a filtered table of the same results showing only non-redundant ontological terms.

**Additional file 7.** Clusters of genes co-expressed between Patient Groups 1 and 4. Normalized read counts from the DESeq2 analysis were used to generate clusters of genes with positively correlated transcription profiles using coseq. 251 DEGs formed six clusters; four clusters with dynamics that clearly distinguish the two patient groups are shown here. Boxplots (left) represent individual patient, shaded by Patient Group; the black line connecting boxplots is the mean expression value of all genes in the cluster. a) MSP-7C is co-expressed with 20 other genes such as PIESP1, PMV, and rhoptry neck protein 3 (RON3, PvP01_1469200). b) MSP-7H is co-expressed with 12 other genes including serine-repeat antigen-1 (SERA, PvP01_0417100), subtilisin-like protease 3 (SUB3, PvP01_1026800), and high molecular weight rhoptry protein 3 (RhopH3, PvP01_0703800). c) A gametocyte-specific marker, gamete release protein (PvP01_0115300), was co-expressed with TRAG28 and PHISTc, but negatively correlated with PvMSP-7H and other invasion related genes. d) Another gametocyte-specific marker, gamete antigen 27/25, was co-expressed with Plasmodium exported protein (PHIST, PvP01_0734900) and tryptophan-rich protein 18 (TRAG18, PvP01_1033900). The line graphs (right) show the expression pattern of each gene in the cluster across all patients, and specific genes are labelled. Pearson’s correlation coefficient (r) for abundance values of MSP-7H and these selected genes are shown.

**Additional file 8.** Clusters of genes co-expressed between Patient Groups 2 and 4. Normalized read counts from the DESeq2 analysis were used to generate clusters of genes with positively correlated transcription profiles using coseq. 351 DEGs formed seven clusters; four clusters with dynamics that clearly distinguish the two patient groups are shown here. Boxplots (left) represent individual patient, shaded by Patient Group; the black line connecting boxplots is the mean expression value of all genes in the cluster. a) Two PvMSP-7H and -7I are co-expressed with 26 other genes including ETRAMP, rhoptry neck protein 12 (RON12, PvP01_0602300), and membrane associated erythrocyte binding-like protein (MAEBL, PvP01_0948400). b) The schizont stage-specific marker SEA1 is co-expressed with 77 other genes such as parasite-infected erythrocyte surface protein (PIESP1, PvP01_0829800) and MOP. c) A gametocyte-specific marker, gamete antigen 27/25, clustered with 31 other genes including Plasmodium exported protein (PHIST, PvP01_0001440) and tryptophan-rich protein (TRAG24, PvP01_1470100). d) Another stage-specific marker, liver stage antigen 3, clustered with 69 other genes such as Plasmodium exported protein (PHIST, PvP01_0119200) and tryptophan-rich protein (TRAG28, PvP01_0000130), but was negatively correlated with PvMSP-7H.

**Additional file 9.** A spreadsheet describing the layout of all 1173 peptides contained on the custom peptide microarray. Peptide map of 13 MSP7 protein sequences elongated with neutral GSGSGSG linkers at the C- and N-terminus and translated into overlapping peptides, with peptide length of 15 amino acids and peptide overlap of 11 amino acids. Green and red peptides denote positive controls.

**Additional file 10.** Fluorescent signal intensity for 236 peptide spots on a custom PvMSP-7 peptide microarray that gave significant responses to human plasma from natural infections. Signal intensity values in infected and naïve plasma are shown for 236 putative B-cell epitopes identified using a custom MSP-7 peptide microarray, assayed with plasma from clinical vivax malaria infections. Plasma were pooled into five age groups. Peptides shown gave significant responses when normalized using LIMMA (p-value adjusted form multiple tests), i.e. the response was significantly greater than the corresponding response from naïve, uninfected patients. For each peptide, the relevant PvMSP-7 paralog is named, and the number of age groups for which the peptide gave a significant response is specified.

## Data Availability

The dataset supporting the conclusions of this article is available from the EMBL-EBI ArrayExpress database, Accession Number E-MTAB-6753; http://www.ebi.ac.uk/arrayexpress/experiments/E-MTAB-6753.

## Abbreviations

CSP: circumsporozoite protein
MSP-7: merozoite surface protein 7
IDC: intraerythrocytic developmental cycle
PfSEA-1A: schizont egress antigen 1
CPM: Read Count per Million bases
SNP: single nucleotide polymorphism
log2FC: log_2_-transformed fold change
DEG: differentially expressed gene
GO: Gene Ontology
RBP: reticulocyte-binding protein
PCA: principal component analysis
PCR: polymerase chain reaction
BSA: bovine serum albumin
PBS: phosphate buffered solution
FDR: false discovery rate
