# Supplementary material for: Clinical expression and antigenic profiles of a Plasmodium vivax vaccine candidate: merozoite surface protein 7 (PvMSP-7)
Source: Malar J. 2019 Jun 13;18:197. doi: 10.1186/s12936-019-2826-7 (PMC6567670; doi:10.1186/s12936-019-2826-7)
Supplement: Supplementary file 2 — Additional file 2. Principal component analysis (PCA) of ten clinical isolates based on transcriptome-wide single nucleotide polymorphisms (SNPs). RNA-seq data for each clinical isolate was mapped to the P. vivax PvPO1 reference genome (5838 genes) and SNPs (N = 42,988) were extracted using the Genome Analysis Toolkit, version 3.7. SNPs were filtered using the linkage disequilibrium pruning method and PLINK version 1.9. A sliding window size of 50 was set across the transcriptome, advancing with steps of five SNPs, and SNPs with a threshold value above 0.5 were removed. Isolate identity and Patient Groups are labelled. Geographical origin is denoted by coloured symbols: red circle (Yala), blue square (Ubon Ratchathani). [file 12936_2019_2826_MOESM2_ESM.pdf]

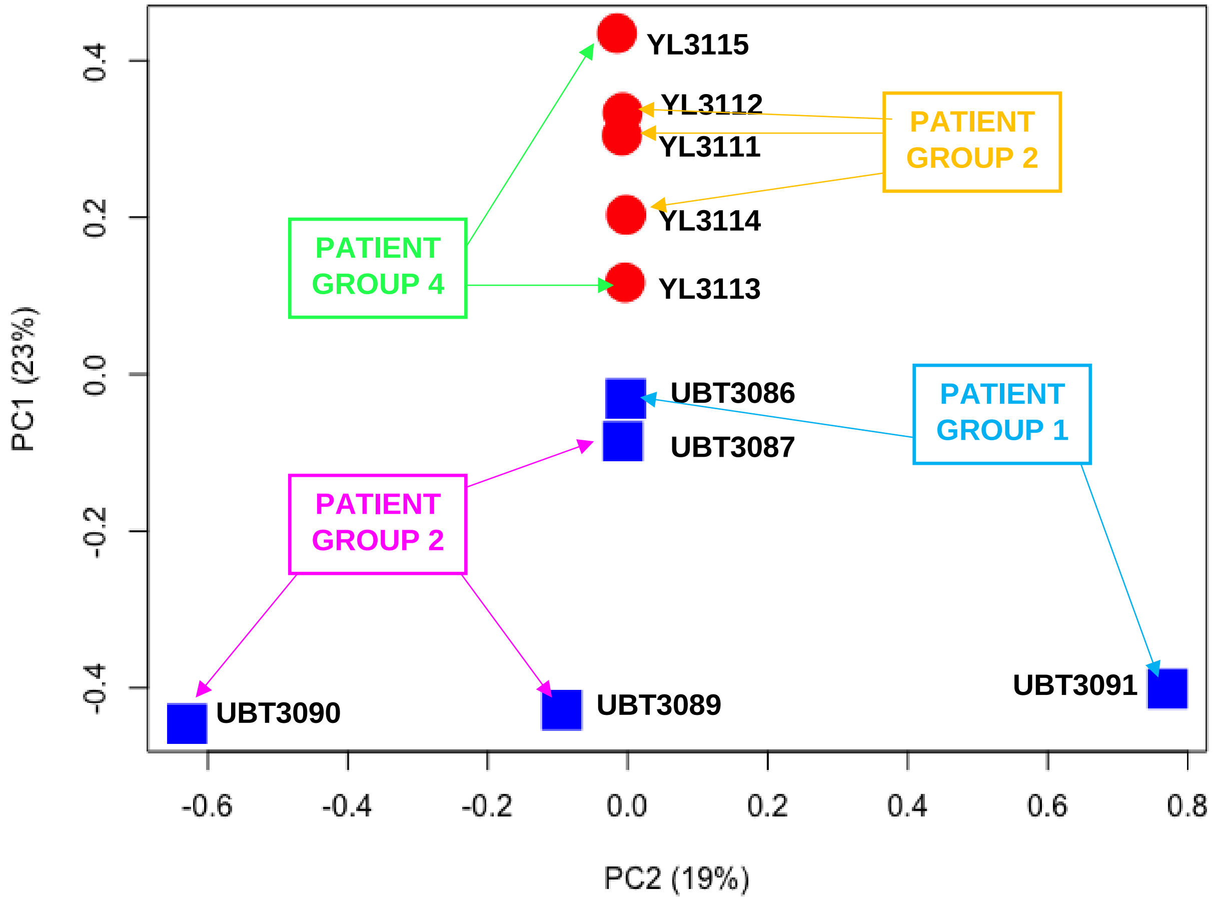

Additional file 2. Principal component analysis (PCA) of ten clinical isolates based on transcriptome-wide single nucleotide polymorphisms (SNPs). RNAseq data for each clinical isolate was mapped to the *P. vivax* PvPO1 reference genome (5, 838 genes) and SNPs (N = 42,988) were extracted using the Genome Analysis Toolkit, version 3.7. SNPs were filtered using the linkage disequilibrium pruning method and PLINK version 1.9. A sliding window size of 50 was set across the transcriptome, advancing with steps of five SNPs, and SNPs with a threshold value above 0.5 were removed. Isolate identity and Patient Groups are labelled. Geogrpahical origin is denoted by coloured symbols: red circle (Yala), blue square (Ubon Ratchathani).
