# Supplementary material for: Clinical expression and antigenic profiles of a Plasmodium vivax vaccine candidate: merozoite surface protein 7 (PvMSP-7)
Source: Malar J. 2019 Jun 13;18:197. doi: 10.1186/s12936-019-2826-7 (PMC6567670; doi:10.1186/s12936-019-2826-7)
Supplement: Supplementary file 4 — Additional file 4. A heatmap describing the profiles of 251 differentially expressed genes (y-axis) between Patient Groups 1 and 4 (x-axis). Data displayed are log2 transformation of normalized raw reads using DESeq2. The heatmap is divided into four sectors. Significant GO terms and/or KEGG pathways in each sector are shown in tables on the right. GO terms and/or KEGG pathways with adjusted p-value<0.05 were deemed significant. The colour scale represents the expression level of DEGs: red (lower expression), black (higher expression). Patient Groups are labelled by colour beneath the dendrogram. [file 12936_2019_2826_MOESM4_ESM.pdf]

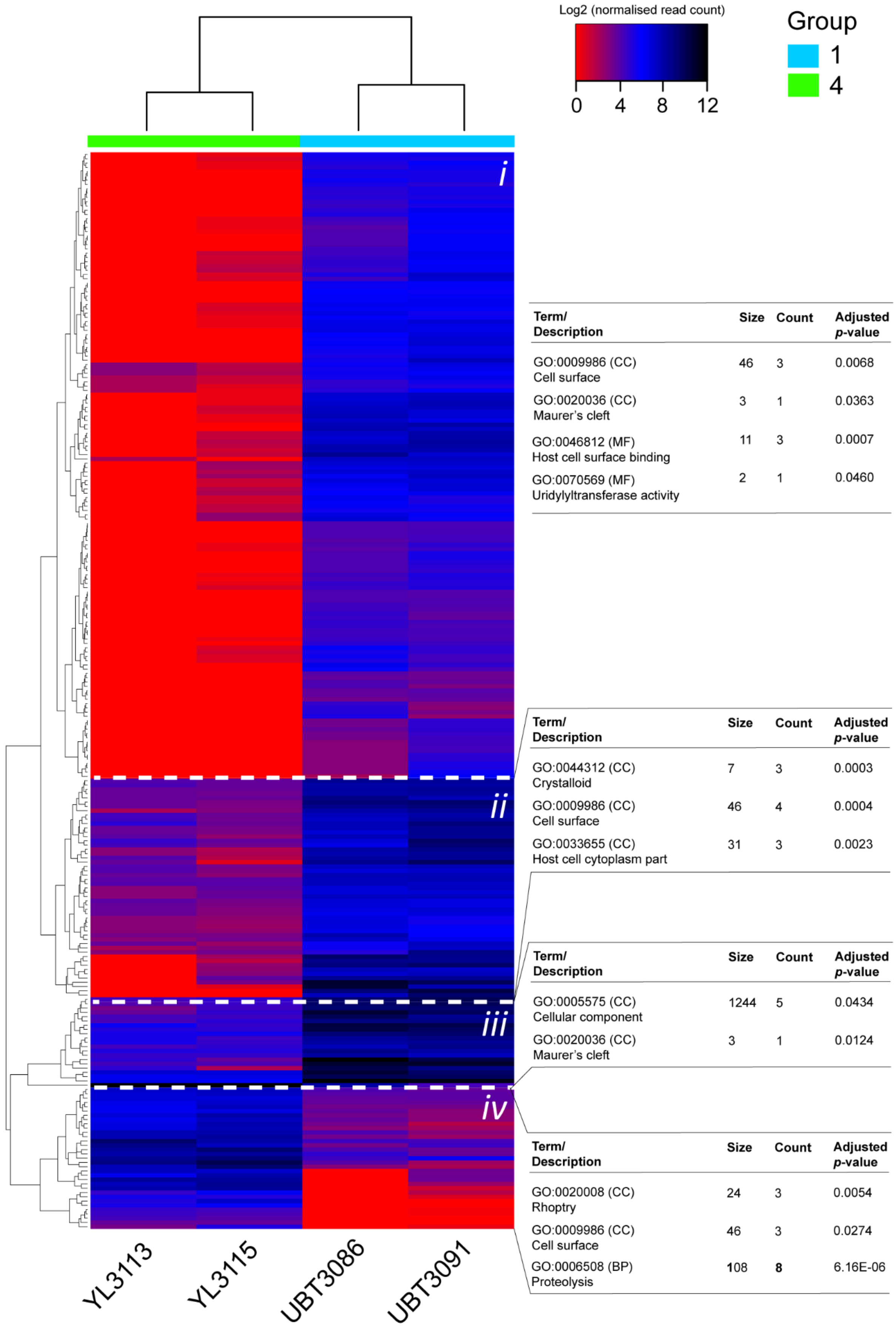

Term/ Description	Size	Count	Adjusted *p*-value
GO:0009986 (CC) Cell surface	46	3	0.0068
GO:0020036 (CC) Maurer's cleft	3	1	0.0363
GO:0046812 (MF) Host cell surface binding	11	3	0.0007
GO:0070569 (MF) Uridylyltransferase activity	2	1	0.0460

Term/ Description	Size	Count	Adjusted *p*-value
GO:0044312 (CC) Crystalloid	7	3	0.0003
GO:0009986 (CC) Cell surface	46	4	0.0004
GO:0033655 (CC) Host cell cytoplasm part	31	3	0.0023

Term/ Description	Size	Count	Adjusted *p*-value
GO:0005575 (CC) Cellular component	1244	5	0.0434
GO:0020036 (CC) Maurer's cleft	3	1	0.0124

Term/ Description	Size	Count	Adjusted *p*-value
GO:0020008 (CC) Rhoptry	24	3	0.0054
GO:0009986 (CC) Cell surface	46	3	0.0274
GO:0006508 (BP) Proteolysis	108	**8**	6.16E-06

Additional file 4. A heatmap describing the profiles of 251 differentially expressed genes (y-axis) between Patient Groups 1 and 4 (x-axis). Data displayed are log_2 transformation of normalised raw reads using *DESeq2*. The heatmap is divided into four sectors. Significant GO terms and/or KEGG pathways in each sector are shown in tables on the right. GO terms and/or KEGG pathways with adjusted *p*-value<0.05 were deemed significant. The colour scale represents the expression level of DEGs: red (lower expression), black (higher expression). Patient Groups are labelled by colour beneath the dendrogram.
