# Supplementary material for: Clinical expression and antigenic profiles of a Plasmodium vivax vaccine candidate: merozoite surface protein 7 (PvMSP-7)
Source: Malar J. 2019 Jun 13;18:197. doi: 10.1186/s12936-019-2826-7 (PMC6567670; doi:10.1186/s12936-019-2826-7)
Supplement: Supplementary file 5 — Additional file 5. A heatmap describing the profiles of 351 differentially expressed genes (y-axis) between Patient Groups 2 and 4 (x-axis). Data displayed are log2 transformation of normalized raw reads using DESeq2. The heatmap is divided into four sectors. Significant GO terms and/or KEGG pathways in each sector are shown in tables on the right. GO terms and/or KEGG pathways with adjusted p-value < 0.05 were deemed significant. The colour scale represents the expression level of DEGs: red (lower expression), black (higher expression). Patient Groups are labelled by colour beneath the dendrogram. [file 12936_2019_2826_MOESM5_ESM.pdf]

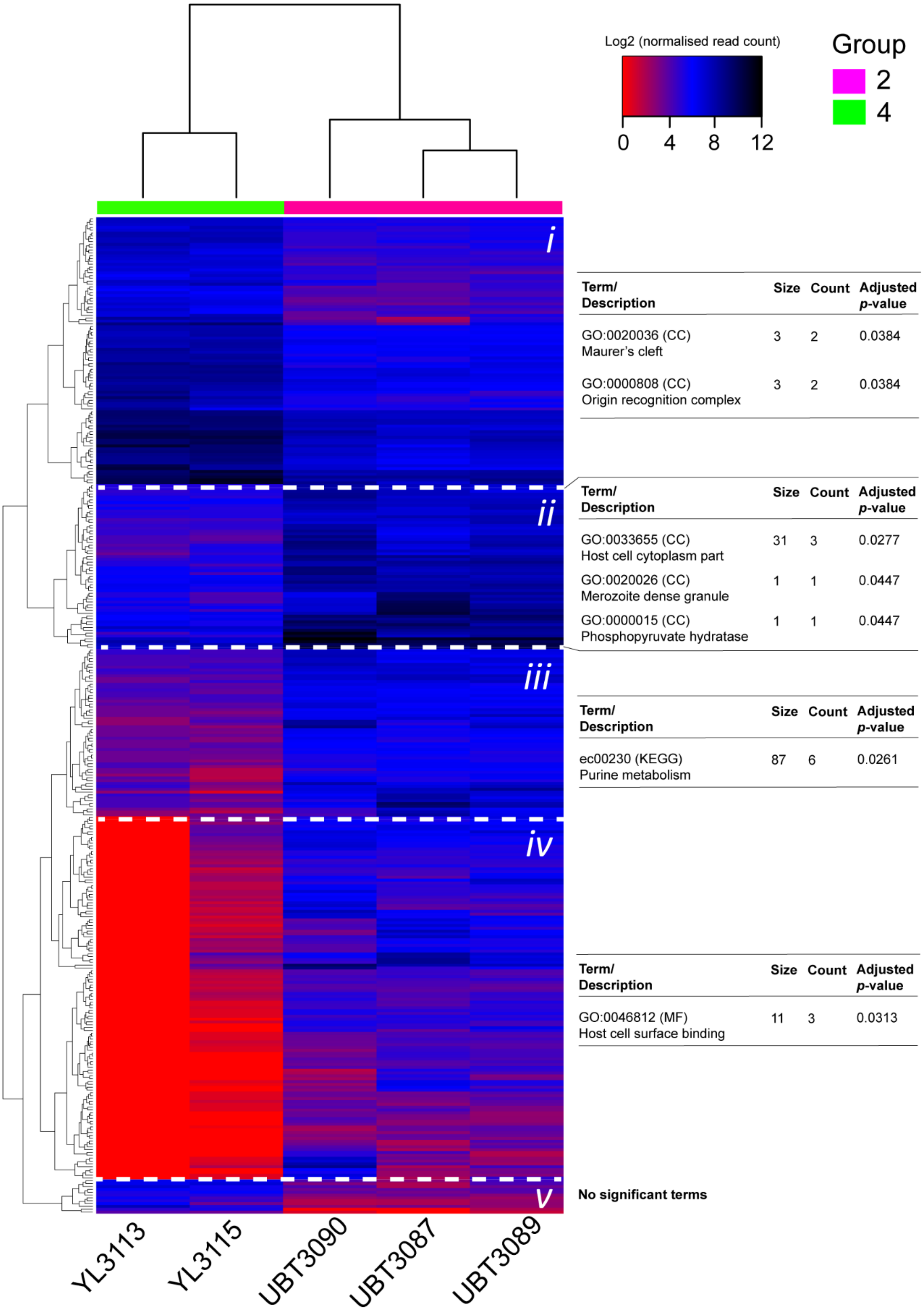

Term/ Description	Size	Count	Adjusted *p*-value
GO:0020036 (CC) Maurer's cleft	3	2	0.0384
GO:0000808 (CC) Origin recognition complex	3	2	0.0384

Term/ Description	Size	Count	Adjusted *p*-value
GO:0033655 (CC) Host cell cytoplasm part	31	3	0.0277
GO:0020026 (CC) Merozoite dense granule	1	1	0.0447
GO:0000015 (CC) Phosphopyruvate hydratase	1	1	0.0447

Term/ Description	Size	Count	Adjusted *p*-value
ec00230 (KEGG) Purine metabolism	87	6	0.0261

Term/ Description	Size	Count	Adjusted *p*-value
GO:0046812 (MF) Host cell surface binding	11	3	0.0313

No significant terms

Additional file 5. A heatmap describing the profiles of 351 differentially expressed genes (y-axis) between Patient Groups 2 and 4 (x-axis). Data displayed are log_2 transformation of normalised raw reads using *DESeq2*. The heatmap is divided into four sectors. Significant GO terms and/or KEGG pathways in each sector are shown in tables on the right. GO terms and/or KEGG pathways with adjusted *p*-value<0.05 were deemed significant. The colour scale represents the expression level of DEGs: red (lower expression), black (higher expression). Patient Groups are labelled by colour beneath the dendrogram.
