# Supplementary material for: Clinical expression and antigenic profiles of a Plasmodium vivax vaccine candidate: merozoite surface protein 7 (PvMSP-7)
Source: Malar J. 2019 Jun 13;18:197. doi: 10.1186/s12936-019-2826-7 (PMC6567670; doi:10.1186/s12936-019-2826-7)
Supplement: Supplementary file 7 — Additional file 7. Clusters of genes co-expressed between Patient Groups 1 and 4. Normalized read counts from the DESeq2 analysis were used to generate clusters of genes with positively correlated transcription profiles using coseq. 251 DEGs formed six clusters; four clusters with dynamics that clearly distinguish the two patient groups are shown here. Boxplots (left) represent individual patient, shaded by Patient Group; the black line connecting boxplots is the mean expression value of all genes in the cluster. a) MSP-7C is co-expressed with 20 other genes such as PIESP1, PMV, and rhoptry neck protein 3 (RON3, PvP01_1469200). b) MSP-7H is co-expressed with 12 other genes including serine-repeat antigen-1 (SERA, PvP01_0417100), subtilisin-like protease 3 (SUB3, PvP01_1026800), and high molecular weight rhoptry protein 3 (RhopH3, PvP01_0703800). c) A gametocyte-specific marker, gamete release protein (PvP01_0115300), was co-expressed with TRAG28 and PHISTc, but negatively correlated with PvMSP-7H and other invasion related genes. d) Another gametocyte-specific marker, gamete antigen 27/25, was co-expressed with Plasmodium exported protein (PHIST, PvP01_0734900) and tryptophan-rich protein 18 (TRAG18, PvP01_1033900). The line graphs (right) show the expression pattern of each gene in the cluster across all patients, and specific genes are labelled. Pearson’s correlation coefficient (r) for abundance values of MSP-7H and these selected genes are shown. [file 12936_2019_2826_MOESM7_ESM.pdf]

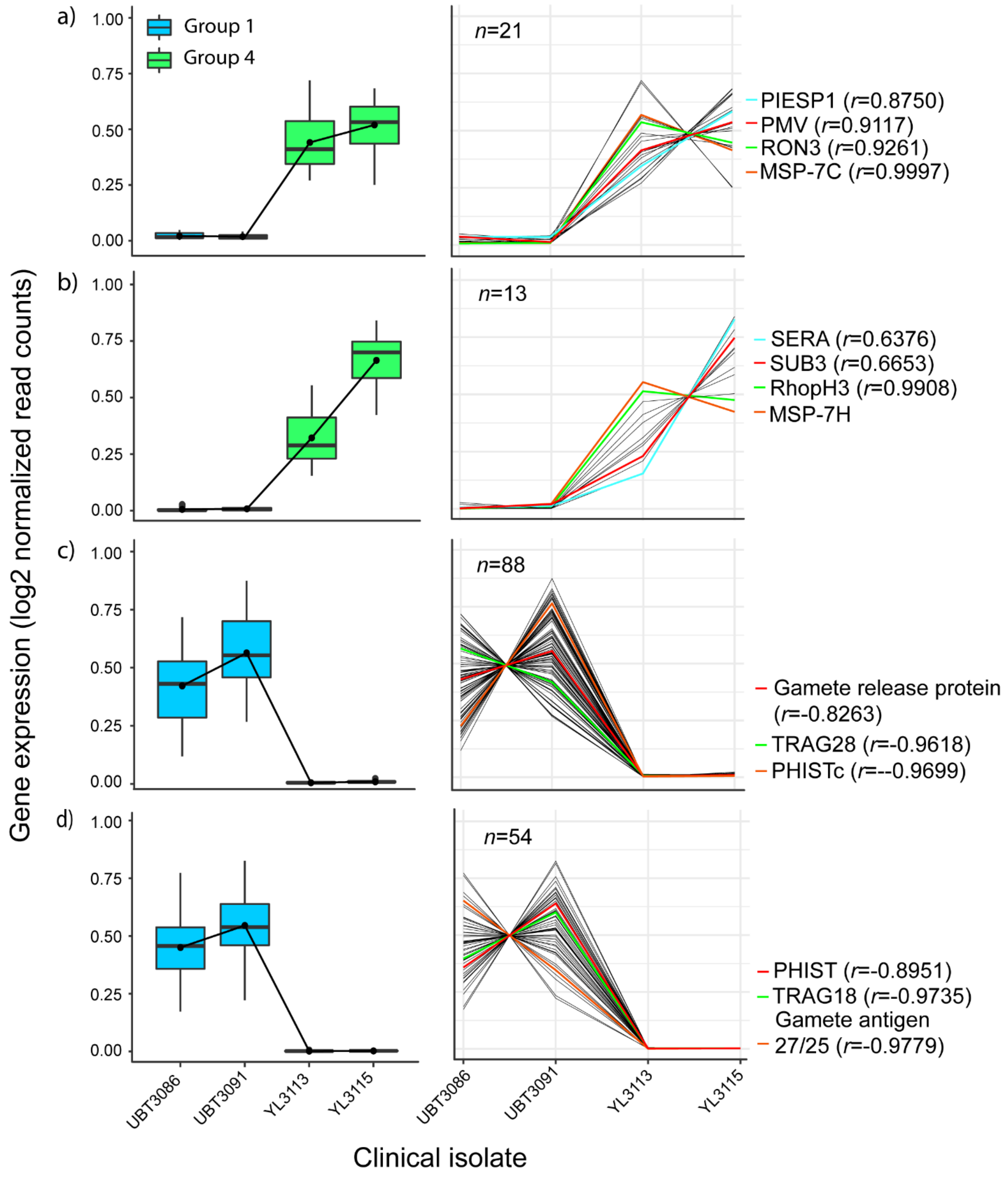

Additional file 7. Clusters of genes co-expressed between Patient Groups 1 and 4. Normalised read counts from the *DESeq2* analysis were used to generate clusters of genes with positively correlated transcription profiles using *coseq*. 251 DEGs formed six clusters; four clusters with dynamics that clearly distinguish the two patient groups are shown here. Boxplots (left) represent individual patient, shaded by Patient Group; the black line connecting boxplots is the mean expression value of all genes in the cluster. **a)** MSP-7C is co-expressed with 20 other genes such as PIESP1, PMV, and rhoptry neck protein 3 (RON3, PvP01_1469200). **b)** MSP-7H is co-expressed with 12 other genes including serine-repeat antigen-1 (SERA, PvP01_0417100), subtilisin-like protease 3 (SUB3, PvP01_1026800), and high molecular weight rhoptry protein 3 (RhopH3, PvP01_0703800). **c)** A gametocyte-specific marker, gamete release protein (PvP01_0115300), was co-expressed with TRAG28 and PHISTc, but negatively correlated with PvMSP-7H and other invasion related genes. **d)** Another gametocyte-specific marker, gamete antigen 27/25, was co-expressed with *Plasmodium* exported protein (PHIST, PvP01_0734900) and tryptophan-rich protein 18 (TRAG18, PvP01_1033900). The line graphs (right) show the expression pattern of each gene in the cluster across all patients, and specific genes are labelled. Pearson's correlation coefficient (*r*) for abundance values of MSP-7H and these selected genes are shown.
