# Supplementary material for: Clinical expression and antigenic profiles of a Plasmodium vivax vaccine candidate: merozoite surface protein 7 (PvMSP-7)
Source: Malar J. 2019 Jun 13;18:197. doi: 10.1186/s12936-019-2826-7 (PMC6567670; doi:10.1186/s12936-019-2826-7)
Supplement: Supplementary file 8 — Additional file 8. Clusters of genes co-expressed between Patient Groups 2 and 4. Normalized read counts from the DESeq2 analysis were used to generate clusters of genes with positively correlated transcription profiles using coseq. 351 DEGs formed seven clusters; four clusters with dynamics that clearly distinguish the two patient groups are shown here. Boxplots (left) represent individual patient, shaded by Patient Group; the black line connecting boxplots is the mean expression value of all genes in the cluster. a) Two PvMSP-7H and -7I are co-expressed with 26 other genes including ETRAMP, rhoptry neck protein 12 (RON12, PvP01_0602300), and membrane associated erythrocyte binding-like protein (MAEBL, PvP01_0948400). b) The schizont stage-specific marker SEA1 is co-expressed with 77 other genes such as parasite-infected erythrocyte surface protein (PIESP1, PvP01_0829800) and MOP. c) A gametocyte-specific marker, gamete antigen 27/25, clustered with 31 other genes including Plasmodium exported protein (PHIST, PvP01_0001440) and tryptophan-rich protein (TRAG24, PvP01_1470100). d) Another stage-specific marker, liver stage antigen 3, clustered with 69 other genes such as Plasmodium exported protein (PHIST, PvP01_0119200) and tryptophan-rich protein (TRAG28, PvP01_0000130), but was negatively correlated with PvMSP-7H. [file 12936_2019_2826_MOESM8_ESM.pdf]

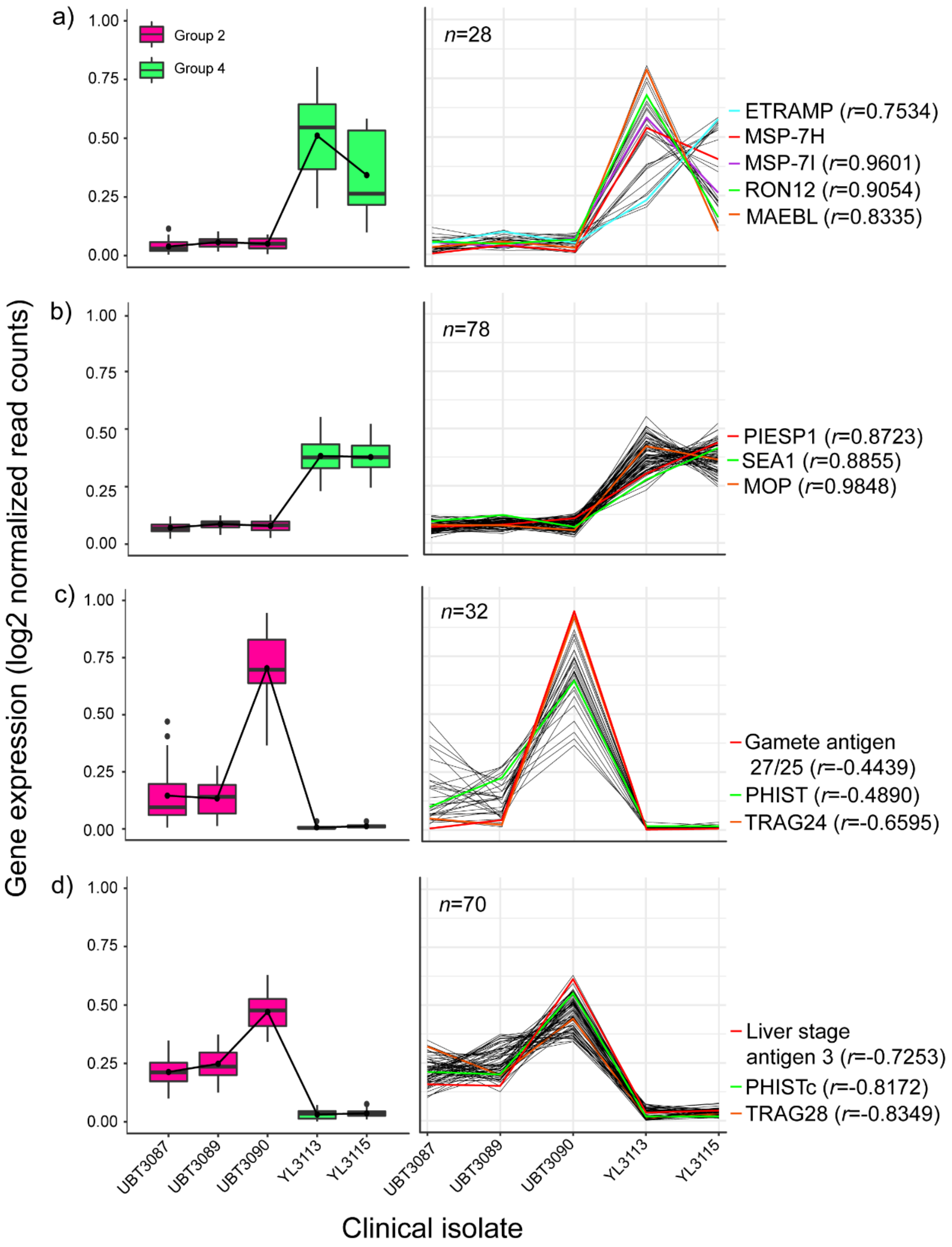

Additional file 8. Clusters of genes co-expressed between Patient Groups 2 and 4. Normalised read counts from the *DESeq2* analysis were used to generate clusters of genes with positively correlated transcription profiles using *coseq*. 351 DEGs formed seven clusters; four clusters with dynamics that clearly distinguish the two patient groups are shown here. Boxplots (left) represent individual patient, shaded by Patient Group; the black line connecting boxplots is the mean expression value of all genes in the cluster. **a)** Two PvMSP-7H and -7I are co-expressed with 26 other genes including ETRAMP, rhoptry neck protein 12 (RON12, PvP01_0602300), and membrane associated erythrocyte binding-like protein (MAEBL, PvP01_0948400). **b)** The schizont stage-specific marker SEA1 is co-expressed with 77 other genes such as parasite-infected erythrocyte surface protein (PIESP1, PvP01_0829800) and MOP. **c)** A gametocyte-specific marker, gamete antigen 27/25, clustered with 31 other genes including *Plasmodium* exported protein (PHIST, PvP01_0001440) and tryptophan-rich protein (TRAG24, PvP01_1470100). **d)** Another stage-specific marker, liver stage antigen 3, clustered with 69 other genes such as *Plasmodium* exported protein (PHIST, PvP01_0119200) and tryptophan-rich protein (TRAG28, PvP01_0000130), but was negatively correlated with PvMSP-7H.
